# Supplementary material for: Novel Selective and Low-Toxic Inhibitor of LmCPB2.8ΔCTE (CPB) One Important Cysteine Protease for Leishmania Virulence
Source: Biomolecules. 2022 Dec 19;12(12):1903. doi: 10.3390/biom12121903 (PMC9775234; doi:10.3390/biom12121903)
Supplement: Supplementary file 1 [file biomolecules-12-01903-s001.zip › biomolecules-2083302-supplementary.pdf]

Supplementary Information

Novel Selective and Low-Toxic Inhibitor of *Lm*CPB2.8ΔCTE (CPB) One Important Cysteine Protease for *Leishmania* Virulence

Vitor Partite Moreira [1], Michele Ferreira da Silva Mela [2], Luana Ribeiro dos Anjos [1], Leonardo Figueiredo Saraiva [3], Angela M. Arenas Velásquez [2], Predrag Kalaba [4], Anna Fabisiková [5], Leandro da Costa Clementino [2], Mohammed Aufy [6], Christian Studenik [6], Natalie Gajic [7], Alexander Prado-Roller [7], Alvicler Magalhães [8], Martin Zehl [5,9], Ingrid Delbone Figueiredo [2], Amanda Martins Baviera [2], Eduardo Maffud Cilli [10], Marcia A. S. Graminha [2,*], Gert Lubec [11,*] and Eduardo R. Perez Gonzalez [1,*]

[1] Fine Organic Chemistry Lab, School of Sciences and Technology, São Paulo State University (UNESP), Presidente Prudente 19060-080, Brazil

[2] School of Pharmaceutical Sciences, São Paulo State University (UNESP), Araraquara 14800-903, Brazil

[3] Laboratory of Luminescence in Materials and Sensors, School of Sciences and Technology, São Paulo State University (UNESP), Presidente Prudente 19060-560, Brazil

[4] Department of Pharmaceutical Sciences, Division of Pharmaceutical Chemistry, Faculty of Life Sciences, University of Vienna, Josef Holaubek Platz 2, UZAII, 1090 Vienna, Austria

[5] Mass Spectrometry Centre, Faculty of Chemistry, University of Vienna, Währinger Straße 38, 1090 Vienna, Austria

[6] Department of Pharmaceutical Sciences, Division of Pharmacology and Toxicology, University of Vienna, Josef Holaubek Platz 2, UZAII (2D 259), 1090 Vienna, Austria

[7] Centre for X-ray Structure Analysis, Faculty of Chemistry, University of Vienna, Währinger Straße 40-42, 1090 Vienna, Austria

[8] Department of Organic Chemistry, Chemistry School, Federal University of Rio de Janeiro, Rio de Janeiro 21941-598, Brazil

[9] Department of Analytical Chemistry, Faculty of Chemistry, University of Vienna, Währinger Straße 38, 1090 Vienna, Austria

[10] Department of Biochemistry and Organic Chemistry, Institute of Chemistry, São Paulo State University (UNESP), Araraquara 14800-060, Brazil

[11] Department of Neuroproteomics, Paracelsus Medical University, 5020 Salzburg, Austria

* Correspondence: marcia.graminha@unesp.br (M.A.S.G.); gert.lubec@lubeclab.com (G.L.); eduardo.gonzalez@unesp.br (E.R.P.G.)

Table of Contents

HRESIMS spectra of LQOF-G6	Page S3-S4
HPLC analysis report LQOF-G6	Page S5
NMR spectra and NMR study of LQOF-G6	Page S6-S15
Characterization data of the new lot of LQOF-G6	Page S15
LC-HPLC analysis report of LQOF-G32	Page S16
ESI-(+)MS and ESI-(+) MS/MS analysis report of LQOF-G32	Page S17
EI-MS analysis report of LQOF-G32.	Page S18
NMR spectra and NMR study of LQOF-G32	Page S18-S20
Characterization data of the new lot of LQOF-G32	Page S20
G6 Crystal data, data collection parameters, and structure refinement	Page S21
Datablock: lqof_g6_pca21.	Page S22-S23
G1 Sample**,** crystal data and data collection and structure refinement.	Page S24
Datablock: prka_lqof_g1_It_p212121.	Page S25-S26
Cell and organ toxicity data	Page S26-S28
Docking Investigation	Page S28-S37
In Vitro Pharmacology: Proteases Assays Report	Page S38-S42

LQOF-G6 Characterization

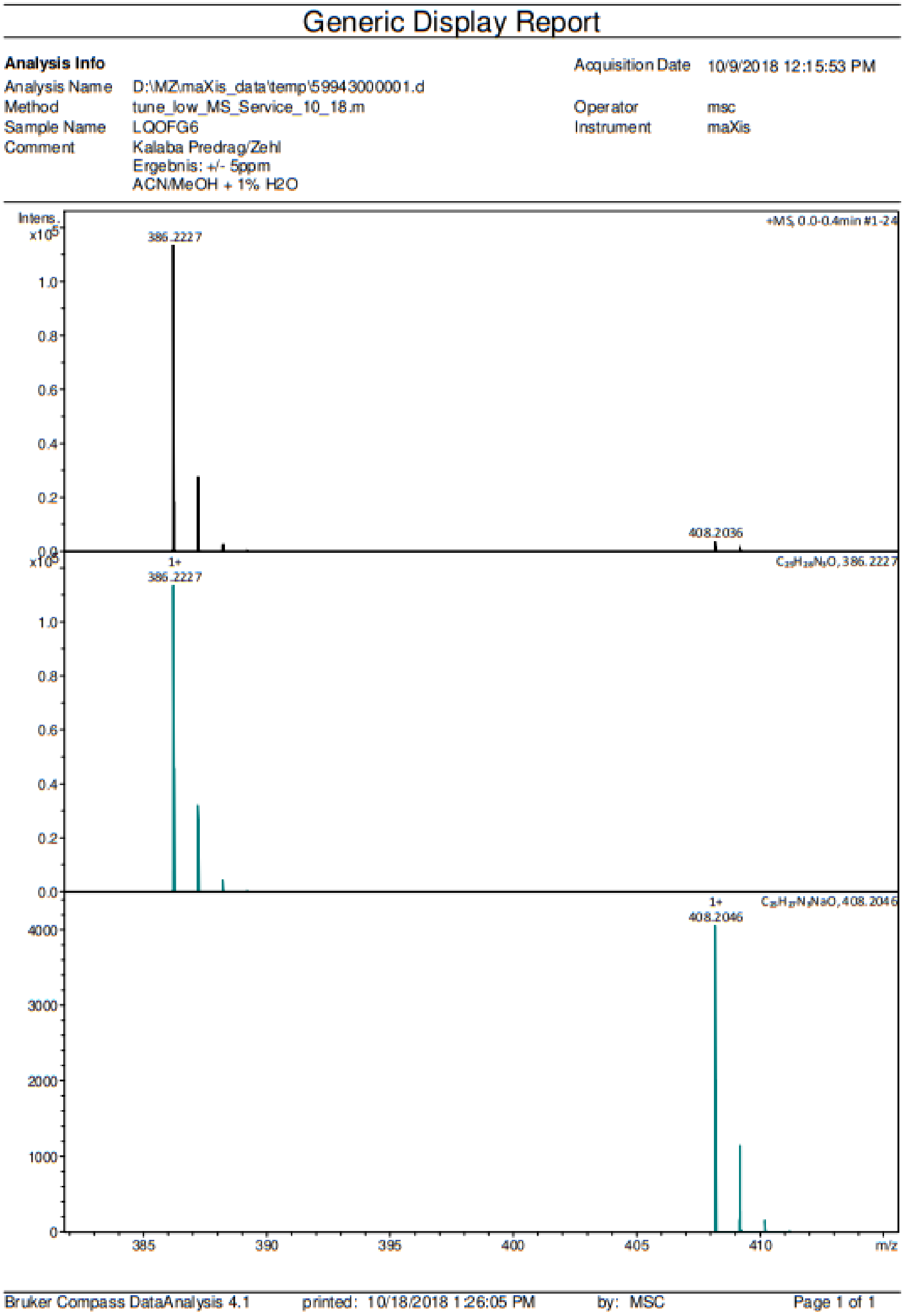

Figure S1. HRESIMS Spectrum of – **LQOF-G6**

Mass Spectrum SmartFormula Report

Analysis Info

Analysis Name	D:\MZ\maXis_data\temp\59943000001.d	Acquisition Date	10/9/2018 12:15:53 PM
Method	tune_low_MS_Service_10_18.m	Operator	msc
Sample Name	LQOFG6	Instrument	maXis 255552.00016
Comment	Kalaba Predrag/Zehl<br>Ergebnis: +/- 5ppm<br>ACN/MeOH + 1% H2O		

Acquisition Parameter

Source Type	ESI	Ion Polarity	Positive	Set Nebulizer	0.4 Bar
Focus	Not active	Set Capillary	4500 V	Set Dry Heater	180 °C
Scan Begin	50 m/z	Set End Plate Offset	-500 V	Set Dry Gas	4.0 l/min
Scan End	1900 m/z	Set Charging Voltage	0 V	Set Divert Valve	Source
		Set Corona	0 nA	Set APCI Heater	0 °C

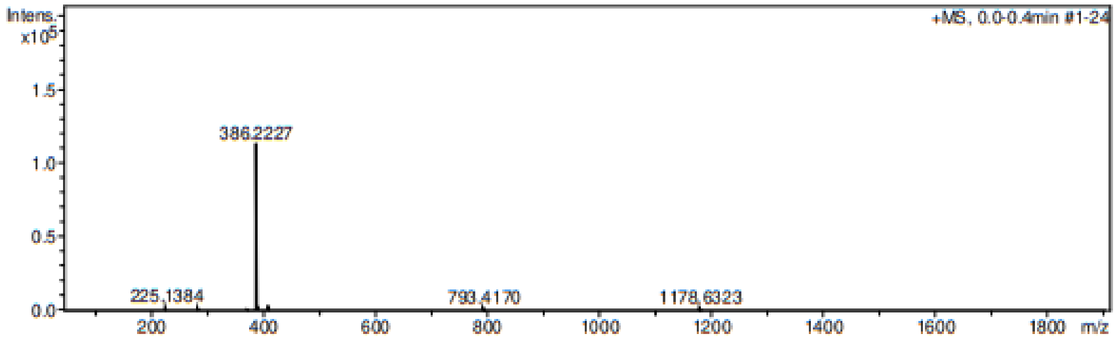

Meas. m/z	#	Ion Formula	Score	m/z	err [mDa]	err [ppm]	mSigma	rdb	e⁻ Conf	N-Rule
386.2227	1	C25H28N3O	100.00	386.2227	-0.0	-0.0	21.7	13.5	even	ok
408.2036	1	C25H27N3NaO	100.00	408.2046	1.1	2.6	25.2	13.5	even	ok

59943000001.d

Bruker Compass DataAnalysis 4.1 printed: 10/18/2018 1:26:10 PM by: MSC Page 1 of 1

Figure S2. HRESIMS Spectrum Smart Formula Report of – **LQOF-G6**

SHIMADZU LabSolutions **Analysis Report**

<Sample Information>

Sample Name	: LQOF-G6		
Sample ID	: LQOF-G6		
Data Filename	: LQOF-G6_purity01.lcd		
Method Filename	: 10-90%_gradient_purity.lcm		
Batch Filename	:		
Vial #	: 1-7	Sample Type	: Unknown
Injection Volume	: 20 uL		
Date Acquired	: 14.12.2018 13:24:54	Acquired by	: System Administrator
Date Processed	: 14.12.2018 14:24:56	Processed by	: System Administrator

<Chromatogram>

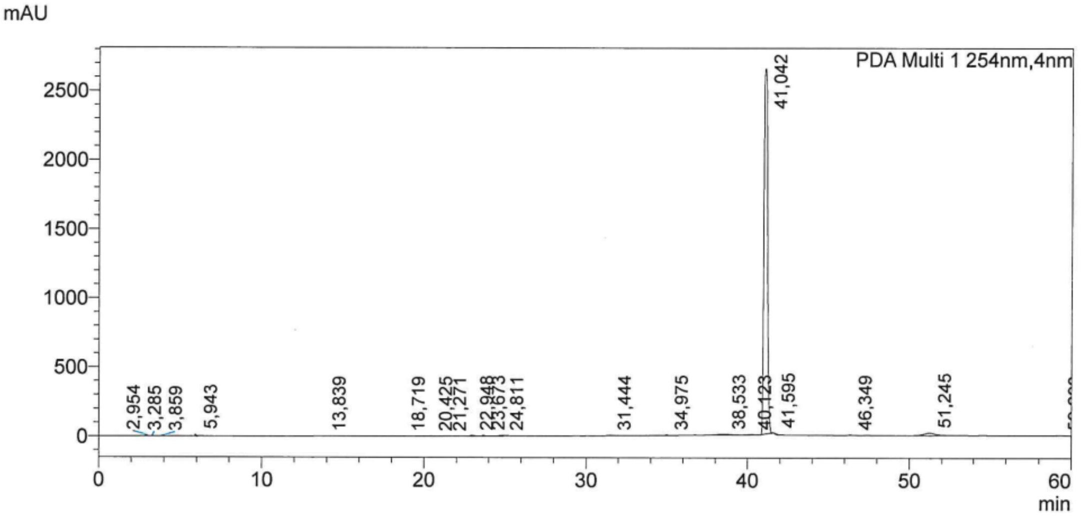

<Peak Table>

PDA Ch1 254nm

Peak#	Ret. Time	Area	Height	Area%
1	2,954	67151	8837	0,158
2	3,285	13046	2712	0,031
3	3,859	1035	311	0,002
4	5,943	23789	12714	0,056
5	13,839	3067	309	0,007
6	18,719	699	189	0,002
7	20,425	2254	551	0,005
8	21,271	3696	687	0,009
9	22,948	54625	4837	0,129
10	23,673	44723	6645	0,105
11	24,811	130190	5109	0,307
12	31,444	80748	3602	0,190
13	34,975	31277	4304	0,074
14	38,533	362108	6594	0,853
15	40,123	73188	2226	0,172
16	41,042	40701752	2642407	95,842
17	41,595	-54767	249	-0,129
18	46,349	32898	2275	0,077
19	51,245	884646	17695	2,083
20	59,293	11432	689	0,027
Total		42467558	2722942	100,000

Figure S3. RP-HPLC analysis report – **LQOF-G6**

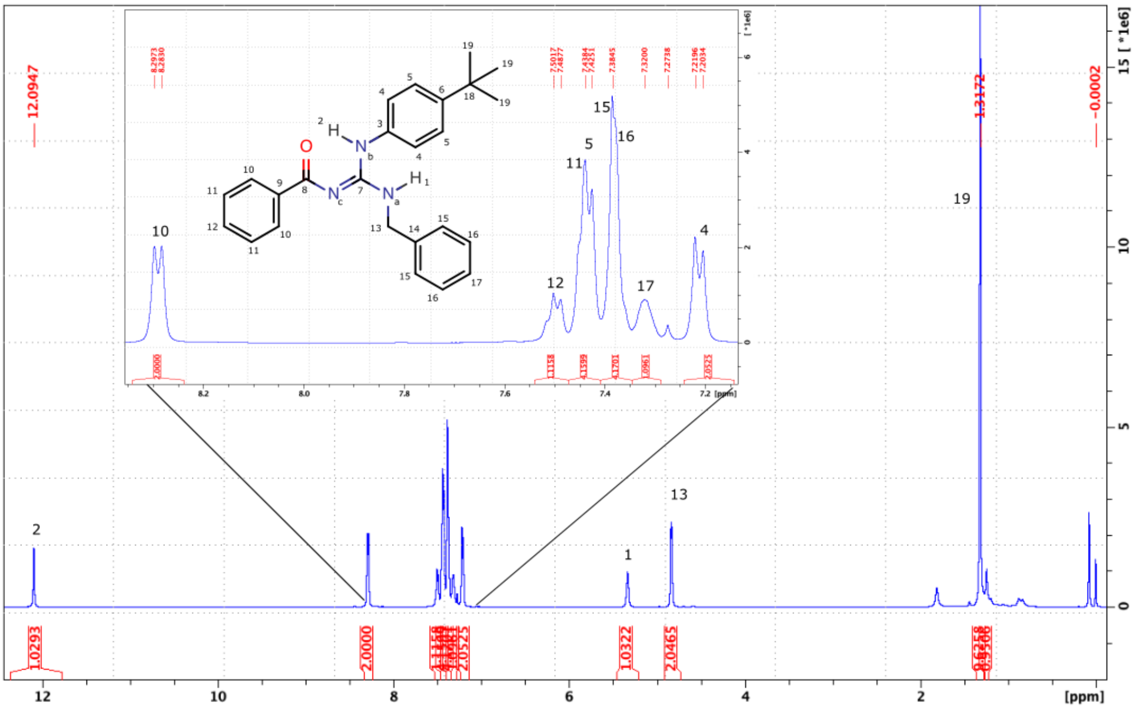

Figure S4. ^{1}H NMR spectrum of **LQOF-G6** at 253K in $CDCl_3$.

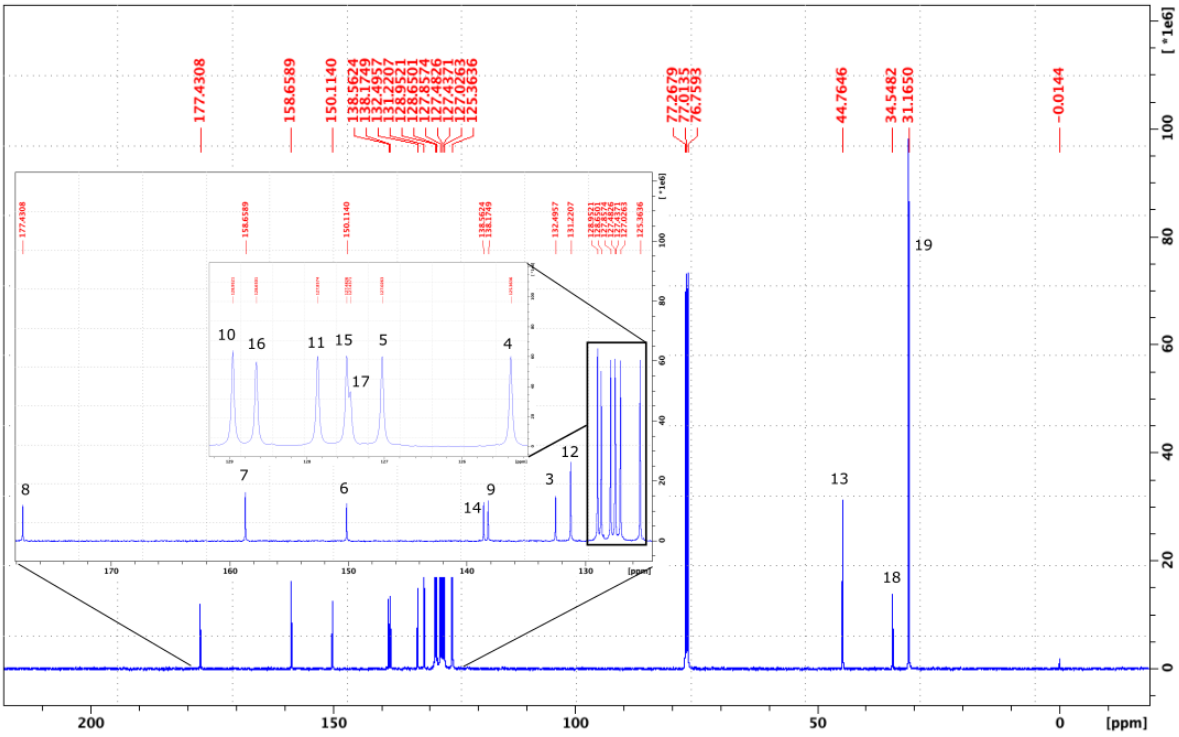

Figure S5. ^{13}C NMR spectrum of **LQOF-G6** at 253K in $CDCl_3$.

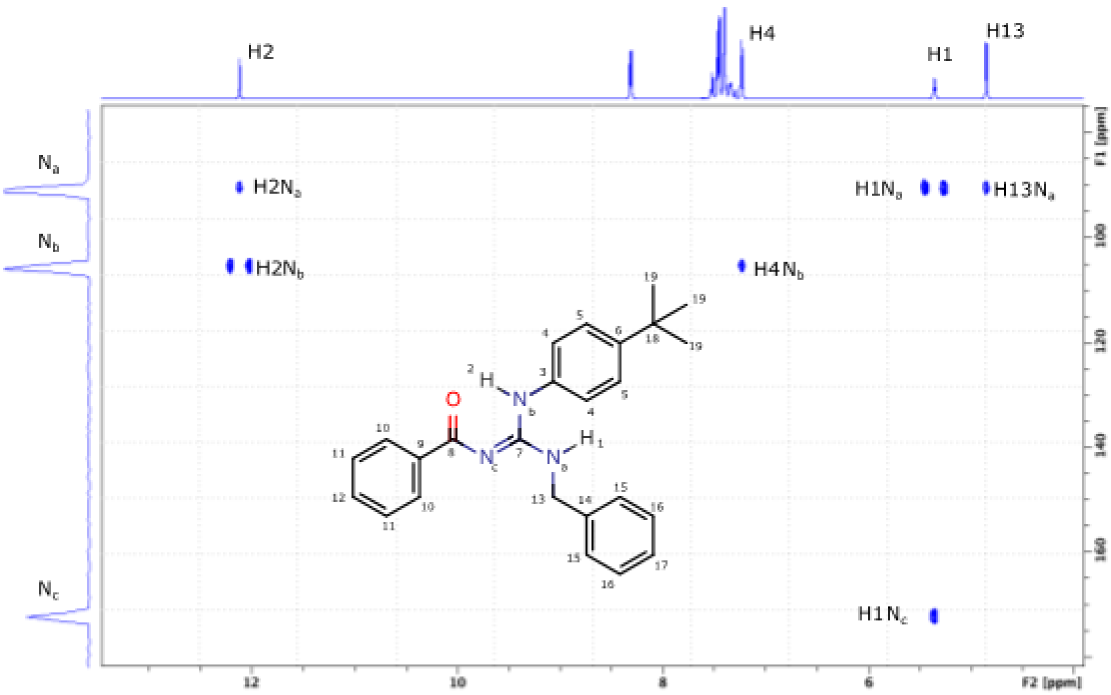

Figure S6. ^{1}H – ^{15}N HMBC spectrum of **LQOF-G6** at 253K.

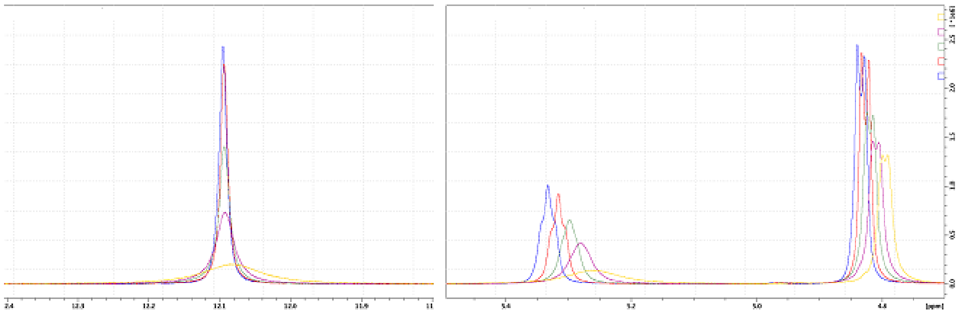

Figure S7. ^{1}H spectrum of **LQOF-G6** in $CDCl_3$ (10 mg/mL) at different temperature values (T(K)= 298 (yellow), 283 (purple), 273 (green), 263 (red) and 253 (blue)). (left) Hydrogen H2. (right) Hydrogens H1 and H13.

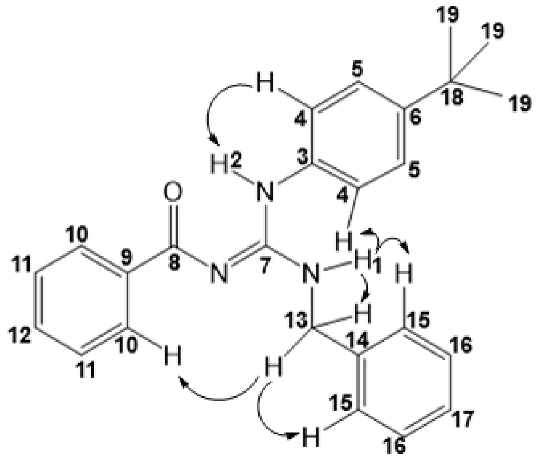

(⟶) **NOESY (253 K)**

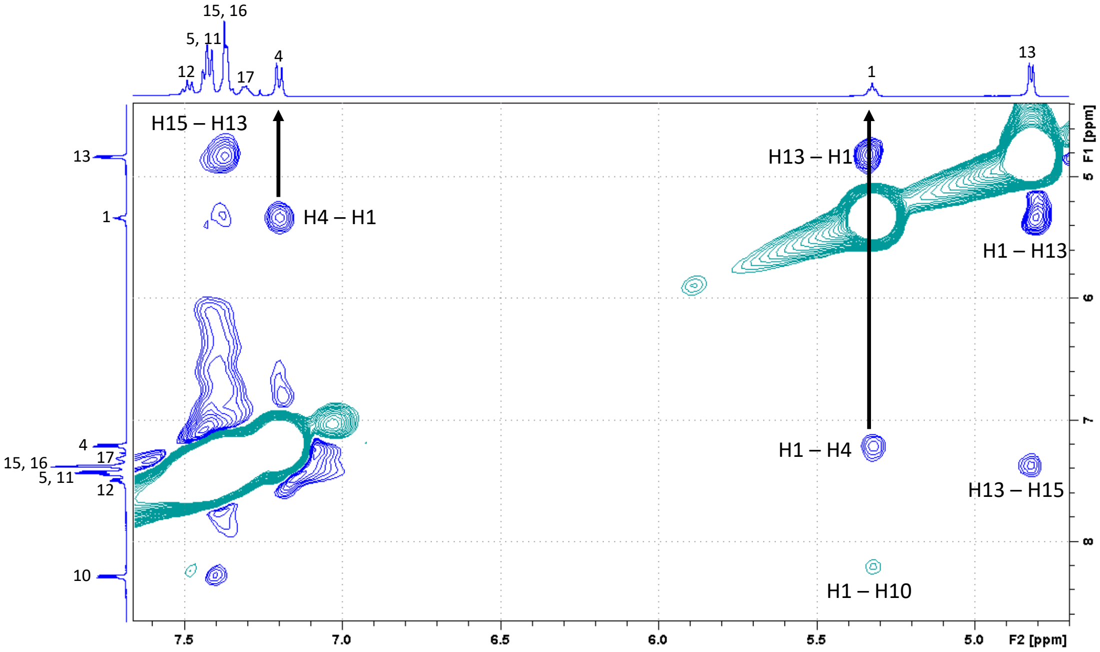

Figure S8. NOESY spectrum in the region of 4.7 to 7.7 ppm, where can be observed the correlation H1-H4 at 253K. The hydrogens shown in the NOESY spectrum correspond to the X-ray output attribution as follows H2(N1A) and H4(C7A).

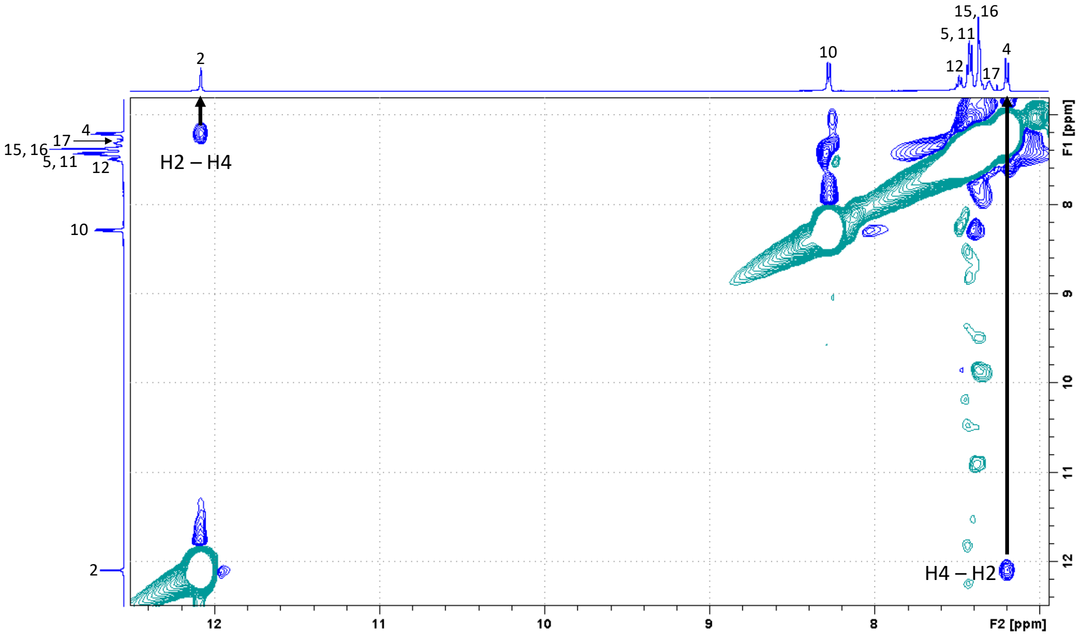

Figure S9. NOESY spectrum in the region of 7.6 to 12.5 ppm, where can be observed the correlation H2-H4 at 253K. The hydrogens shown in the NOESY spectrum correspond to the X-ray output attribution as follows H2(N2A) and H4(C7A).

NMR study

The **LQOF-G6** molecule was characterized in detail by 1D- and 2D-NMR analyses. **Tables S1** and **S2** summarize the values of the chemical shift of the ^{1}H and ^{13}C nuclei of **LQOF-G6** in $CDCl_3$ at 253K. The ^{1}H NMR spectrum (**Figure S3**) shows the signals of the nine equivalent H of the *tert*-butyl group (H19), two methylene (H13), two equivalent aromatic (H10), and two exchangeable *N*-bound hydrogens (H1 and H2). The seven signals of the remaining 12 aromatic hydrogens were clustered in the region 7.20-7.55 ppm with some of them overlapping. The signal at 177.431 ppm in the ^{13}C spectrum at 253K (**Figure S4**) is characteristic for a carbonyl and thus assigned to C8. The signals in the aliphatic region at 44.765, 34.548, and 31.165 ppm were attributed to the benzylic C13, the quaternary C18 and the three equivalent methyl carbons of the *tert*-butyl group C19, respectively.

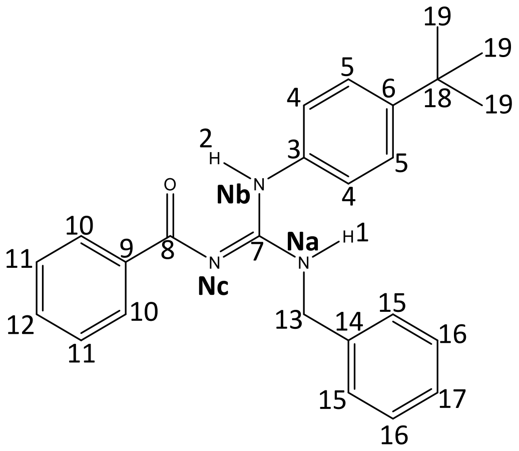

The **LQOF-G6** molecule was characterized in detail by 1D- and 2D-NMR analyses. **Tables S1** and **S2** summarize the values of the chemical shift of the ^{1}H and ^{13}C nuclei of **LQOF-G6** in $CDCl_3$ at 253K. The ^{1}H NMR spectrum (**Figure S4**) shows the signals of the nine equivalent H of the *tert*-butyl group (H19), two methylene (H13), two equivalent aromatic (H10), and two exchangeable *N*-bound hydrogens (H1 and H2). The seven signals of the remaining 12 aromatic hydrogens were clustered in the region 7.20-7.55 ppm with some of them overlapping. The signal at 177.431 ppm in the ^{13}C spectrum at 253K (**Figure S5**) is characteristic for a carbonyl and thus assigned to C8. The signals in the aliphatic region at 44.765, 34.548, and 31.165 ppm were attributed to the benzylic C13, the quaternary C18 and the three equivalent methyl carbons of the *tert*-butyl group C19, respectively.

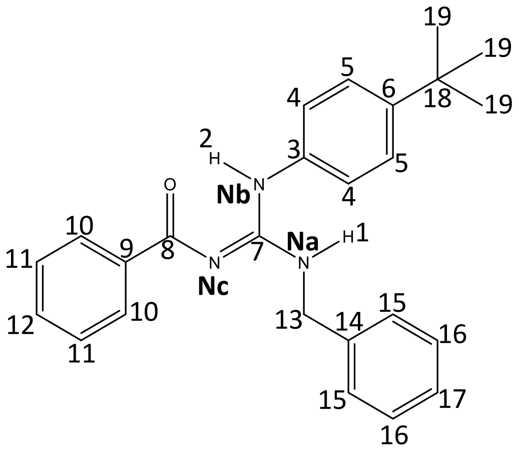

Table S1. [1]H chemical shift values (δ ppm) of the compound **LQOFG-6** in $CDCl_3$ at 253 K.

Atom	H1	H2	H4	H5	H10	H11
δ (ppm)	5.33	12.09	7.21	7.42	8.29	7.43
Multiplicity	T	s	d	d	dd	dt
J (Hz)	5.89		8.26	8.26	7.26/1.11	7.26/7.32
Atom	H12	H13	H15	H16	H17	H19
δ (ppm)	7.50	4.83	7.38	7.37	7.31	1.32
Multiplicity	Tt	d	-	-	-	-
J (Hz)	7.32/1.11	5.89	-	-	-	-

Table S2. [13]C chemical shift values (δ ppm) of the compound **LQOF-G6** in $CDCl_3$ at 253 K.

C3	C4	C5	C6	C7	C8	C9	C10	C11
132.495	125.363	127.026	150.114	158.658	177.430	138.174	128.952	127.857
C12	C13	C14	C15	C16	C17	C18	C19	
131.220	44.764	138.562	127.482	128.650	127.437	34.548	31.165	

As suggest by the chemical shift of 12.09 ppm, H2 is involved in formation of an intramolecular hydrogen bond with the carbonyl group as is the case in the crystal structure. The ^{13}C signal corresponding to C7 appeared at 158.659 ppm, characteristic of a guanidinic nucleus central carbon atom.

For a more complete characterization of **LQOF-G6**, 2D analysis of heteronuclear couplings were performed. Therefore, the assignment of the ^{1}H and ^{13}C signals (**Figures S3** and **S4**) was also confirmed by the corresponding correlations in the ^{1}H-^{13}C HSQC spectrum. The aliphatic region (**Figure S7**) showed signals corresponding to correlations between carbon atoms C19 (31.165 ppm) and C13 (44.765 ppm) with hydrogens H19 (1.32 ppm) and H13 (4.83 ppm), respectively. The aromatic region of the HSQC spectrum (**Figure S8**) shows the following linking of the carbons to their directly bound hydrogens: C4/H4 (125.364/7.21 ppm), C5/H5 (127.026/7.42 ppm), C10/H10 (128.952/8.29 ppm), C12/H12 (131.221/7.50 ppm) and C15/H15 (127.483/7.38 ppm).

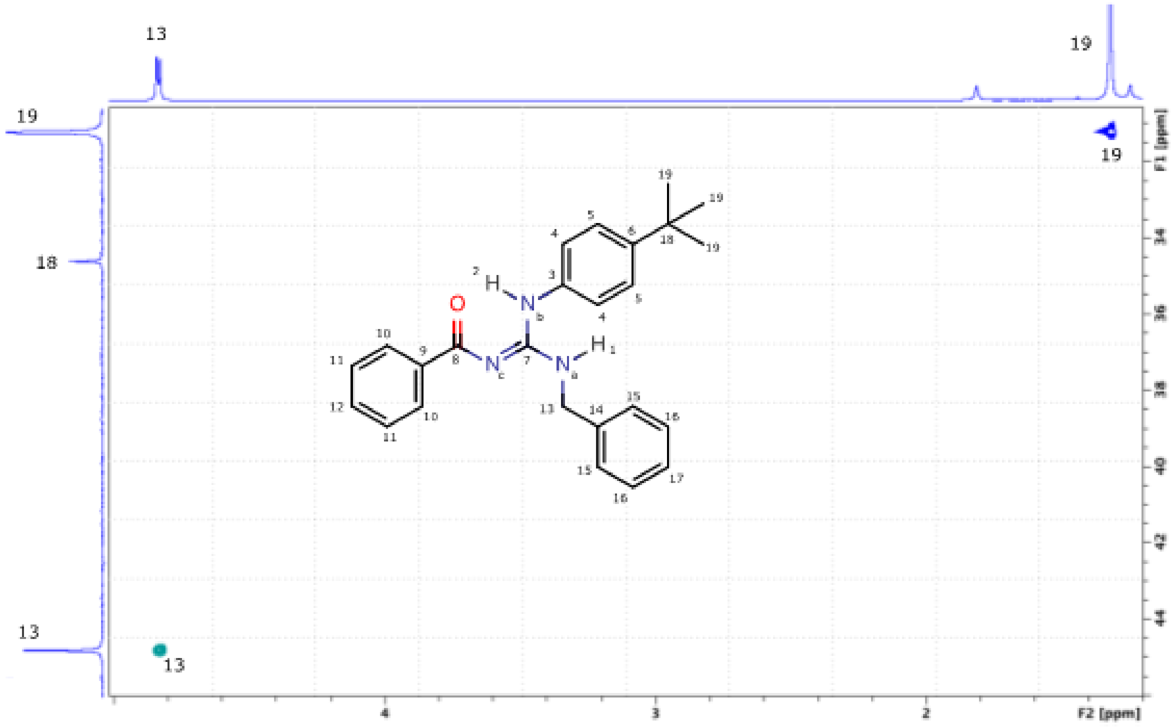

Figure S10. Aliphatic region of the **^{1}H-^{13}C** HSQC spectrum of **LQOF-G6** at 253K in $CDCl_3$.

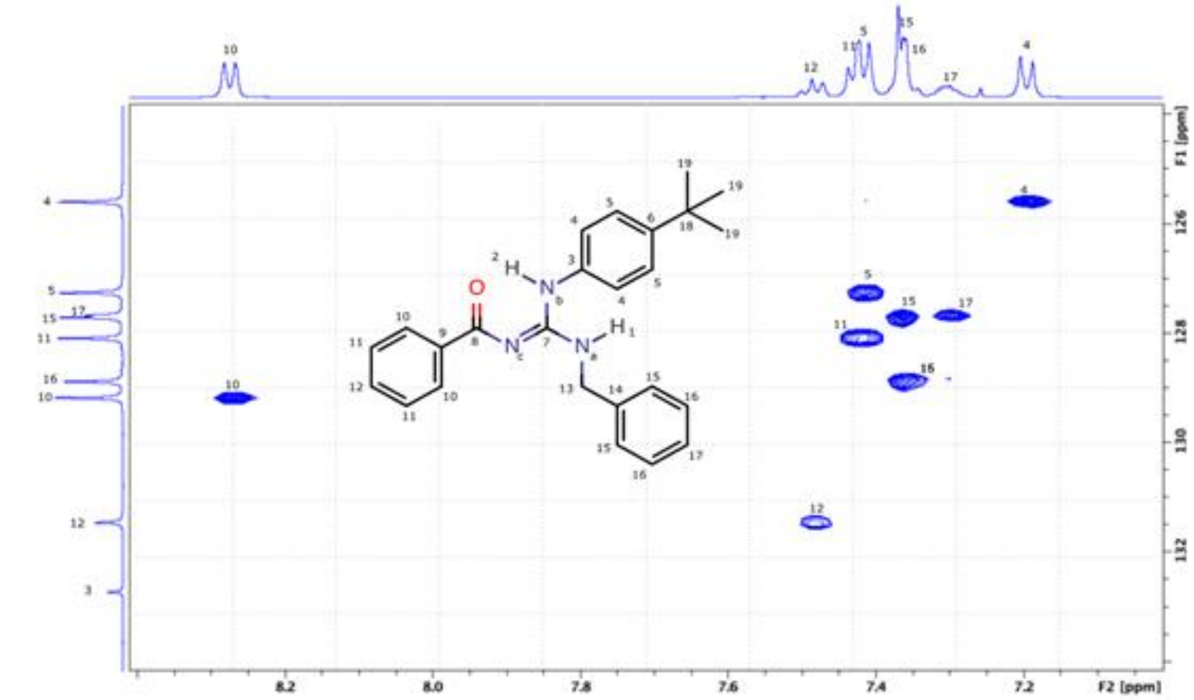

Figure S11. Aromatic region of the ^{1}H-^{13}C HSQC spectrum of **LQOF-G6** at 253K in $CDCl_3$.

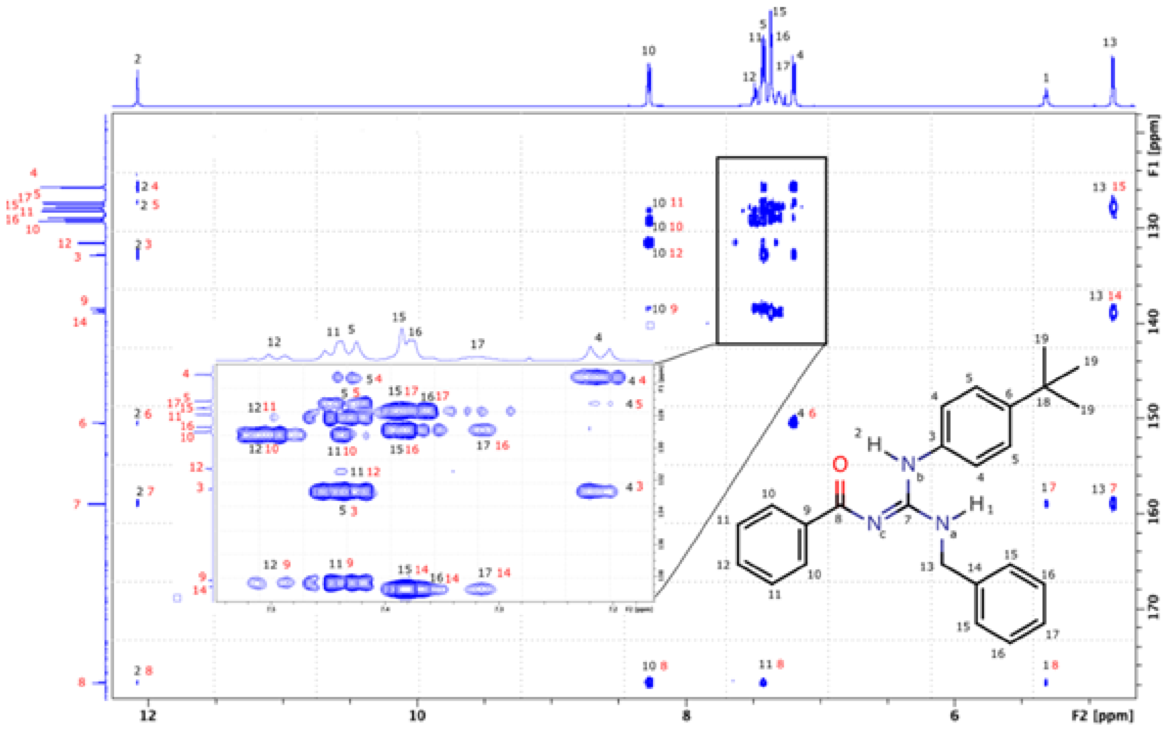

Figure S12. Aromatic region of the ^{1}H-^{13}C HMBC spectrum of **LQOF-G6** at 253K in $CDCl_3$ (black numbers indicate ^{1}H and red numbers indicate ^{13}C).

The ^{1}H-^{13}C HMBC spectrum shows a long-range correlation between H10 at 8.29 ppm and C8 at 177.431 ppm (**Figure S9**), confirming the position of H10 in the benzoyl moiety. Analogously, a strong cross-peak between the *tert*-butyl group H19 (1.31 ppm) and the quaternary C6 at 150.114 ppm positions the latter in the aniline ring. H4 (7.21 ppm) also displays coupling with C6 and H5 (7.42 ppm), which couples with C3 (132.496 ppm), is identified as adjacent to H4. Furthermore, long-distance ^{1}H-^{13}C heteronuclear couplings were observed for H10 (8.29 ppm) with C11 (127.857 ppm) and C12 (131.221 ppm), of H12 (7.50 ppm) with C11, and H11 (7.43 ppm) with C9 (138.175 ppm) within the benzoyl group as well as of H15/H16 (7.38/7.37 ppm) with C13 (45.765 ppm) and C14 (138.562 ppm), of H13 (4.83 ppm) with C14 and C15 (127.483 ppm) within the benzyl group. The central C7 (158.676 ppm) showed correlation signals from H1 (5.33 ppm), H2 (12.09 ppm), and H13 (4.83 ppm). **Figure S10** shows the most representative HMBC interactions for **LQOF-G6**. All these attributed signals support the successful formation of the **LQOF-G6** guanidine molecule.

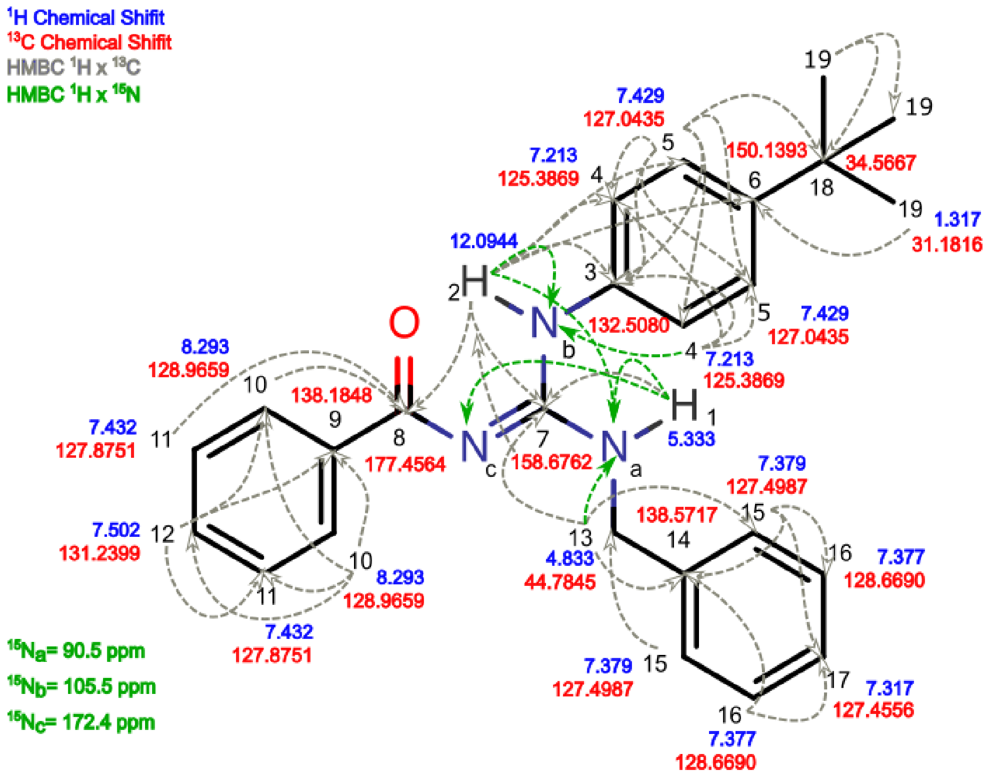

Figure S13. HMBC interactions of **LQOF-G6**.

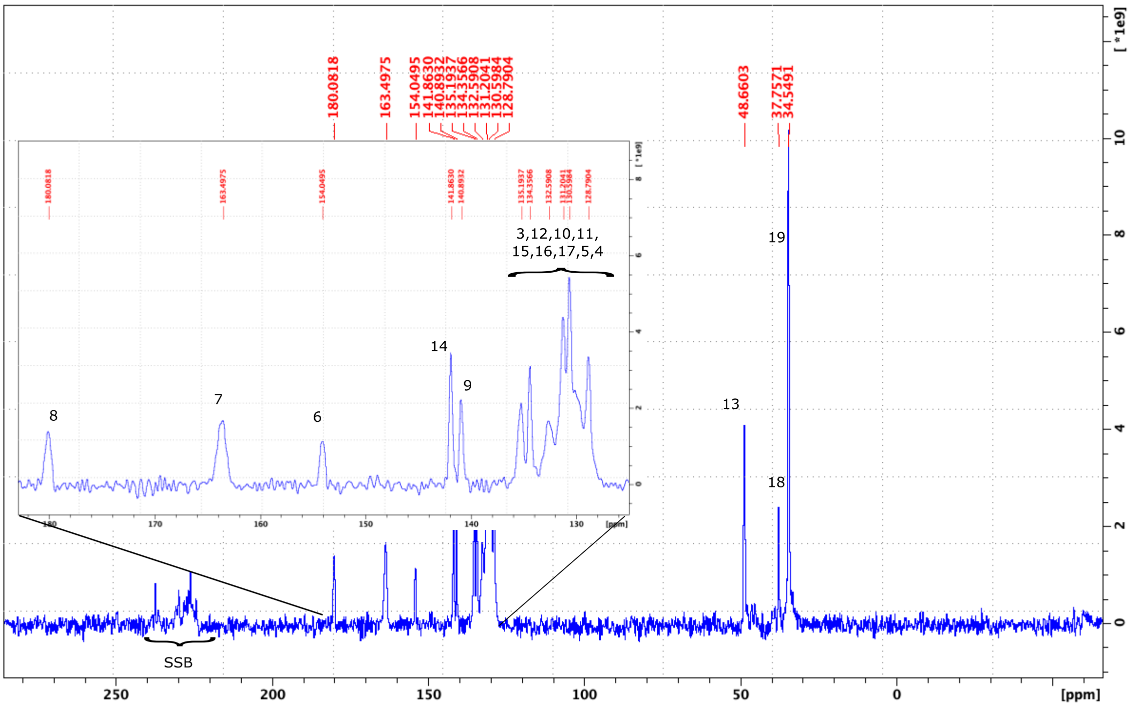

Figure S14. Cross polarization $^{13}C\{^{1}H\}$ solid state NMR spectra of **LQOF-G6**. Contact time 3 ms during polarization ramp 50 to 100%, spun at 12 kHz, SSB - indicate spin side band, ^{13}C acquired at 125.695 MHz, ^{1}H at 499.832 MHz was decoupled using 70 kHz pulse power with spinal 64 sequence.

LQOF-G6 new batch characterization data: ^{1}H NMR (500.13 MHz, $CDCl_3$) δ = 12.09 (s , 1H), 8.29 (dd, *J* = 7.26/1.11 Hz, 2H), 7.50 (tt, *J* = 7.32/1.11 Hz, 1H), 7.43 (dt, , *J* = 7.26/7.32 Hz, 2H), 7.42 (d, *J* = 8.26 Hz, 2H), 7.37 (2H), 7.317 (1H), 7.21 (d, *J* = 8.26 Hz, 2H), 5,33 (t, *J* = 5.89 Hz, 1H), 4.83 (d, *J* = 5.89 Hz, 2H), 1.31 (9H); ^{13}C NMR (100.61 MHz, $CDCl_3$) δ = 177.456 (C=O), 158.676 (N=C), 150.139 (C), 138.571 (C), 138.184 (C), 133.508 (C), 131.239 (*p*-CH), 128.965 (2CH), 128.669 (2CH), 127.875 (2CH), 127.498 (2CH + *p*-CH), 127.455 (2CH), 127.043 (2CH), 125.386 (2CH), 45.784 (N-CH_2), 34.566 (C), 31.181 (CH_3). HRESIMS m/z 386.2227 $[M+H]^+$ (calcd. for $C_{25}H_{28}N_3O^+$, 386.2227, Δ = - 0.0 ppm); HPLC purity 95.8%; mp. 124.4-125.7 ºC.

LQOF-G32 Characterization

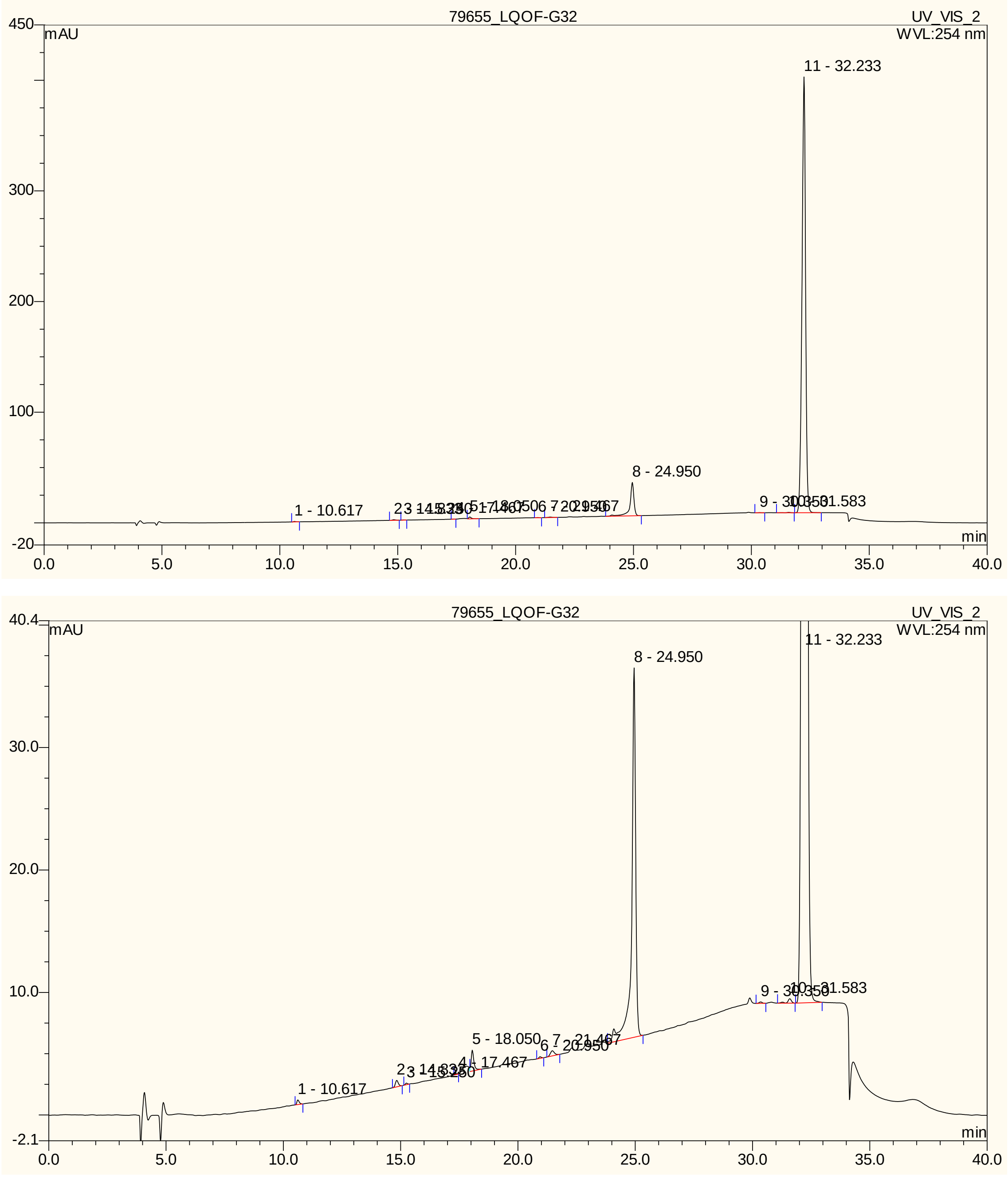

Figura S15. LC-(HPLC) analysis report – **LQOF-G32**

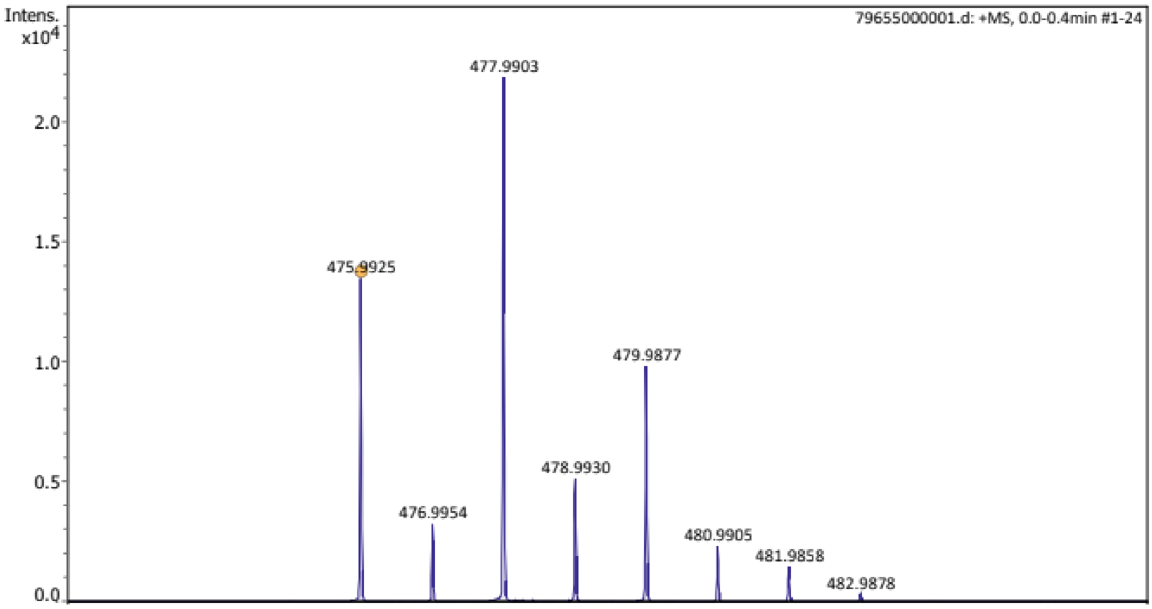

Figure S16. ESI-(+)MS analysis report of LQOF-G32

.

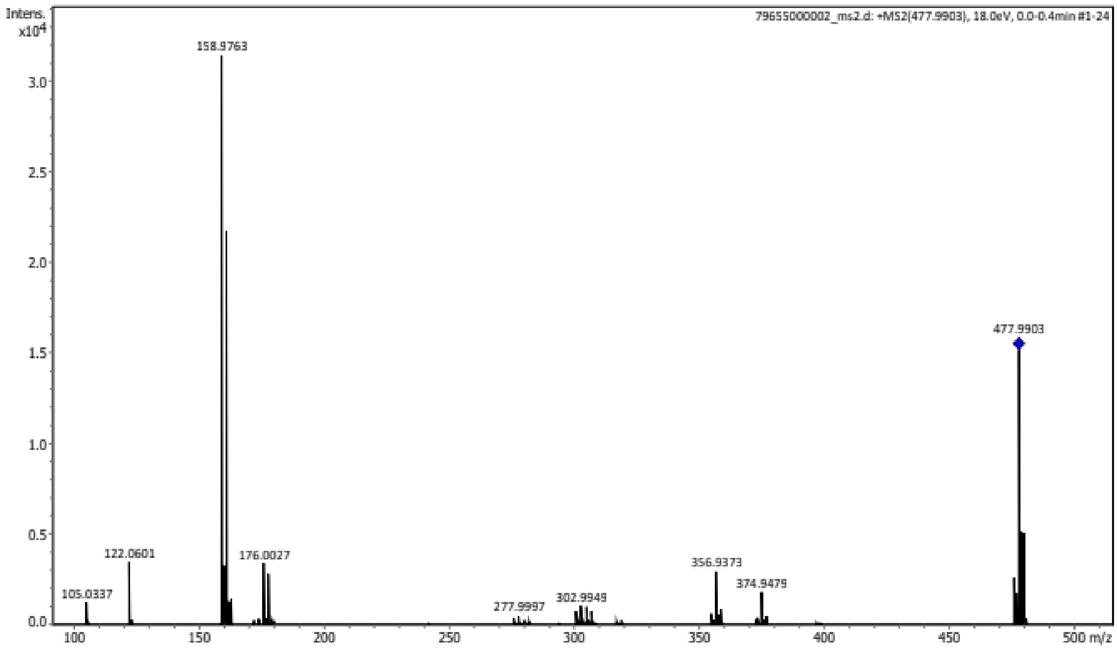

Figure S17. ESI-(+) MS/MS analysis report of LQOF-G32.

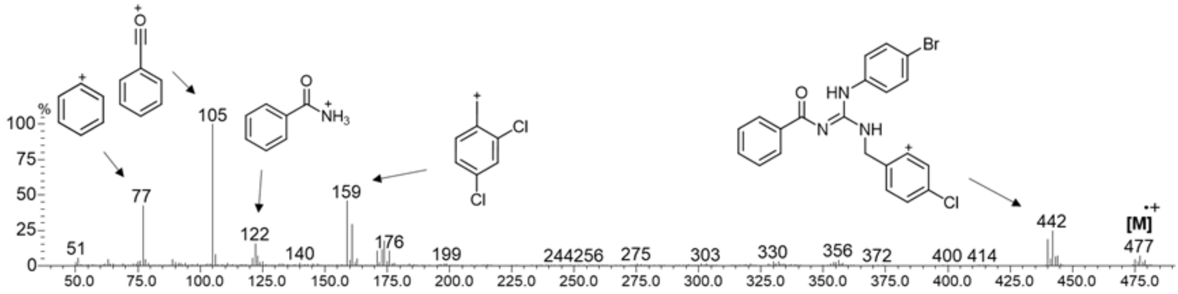

Figure S18. EI-MS analysis report of LQOF-G32.

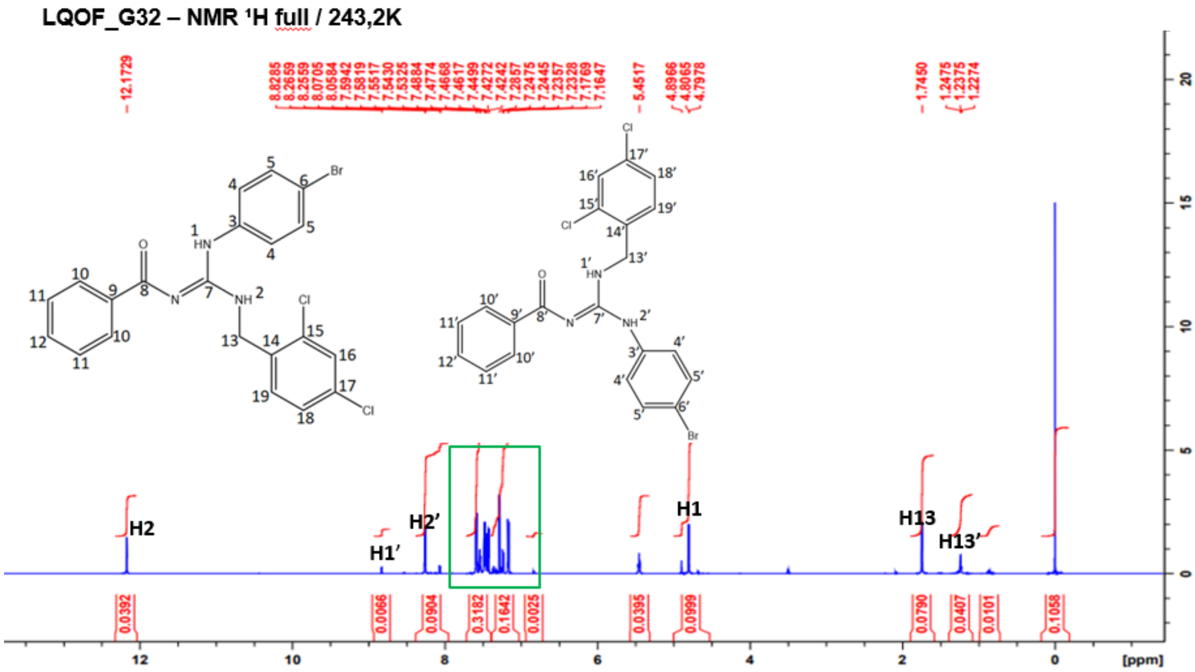

Figure S19. ^{1}H NMR full spectrum of **LQOF-G32** at 243K in $CDCl_3$.

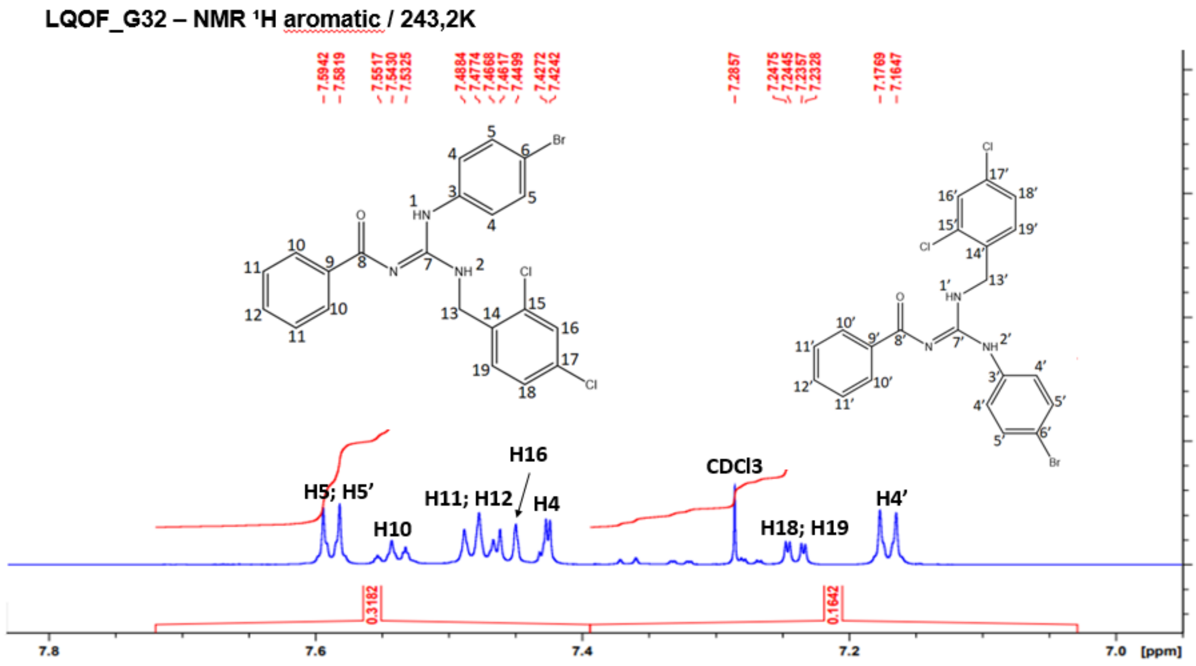

Figure S20. ^{1}H NMR aromatic spectrum of **LQOF-G32** at 243K in $CDCl_3$.

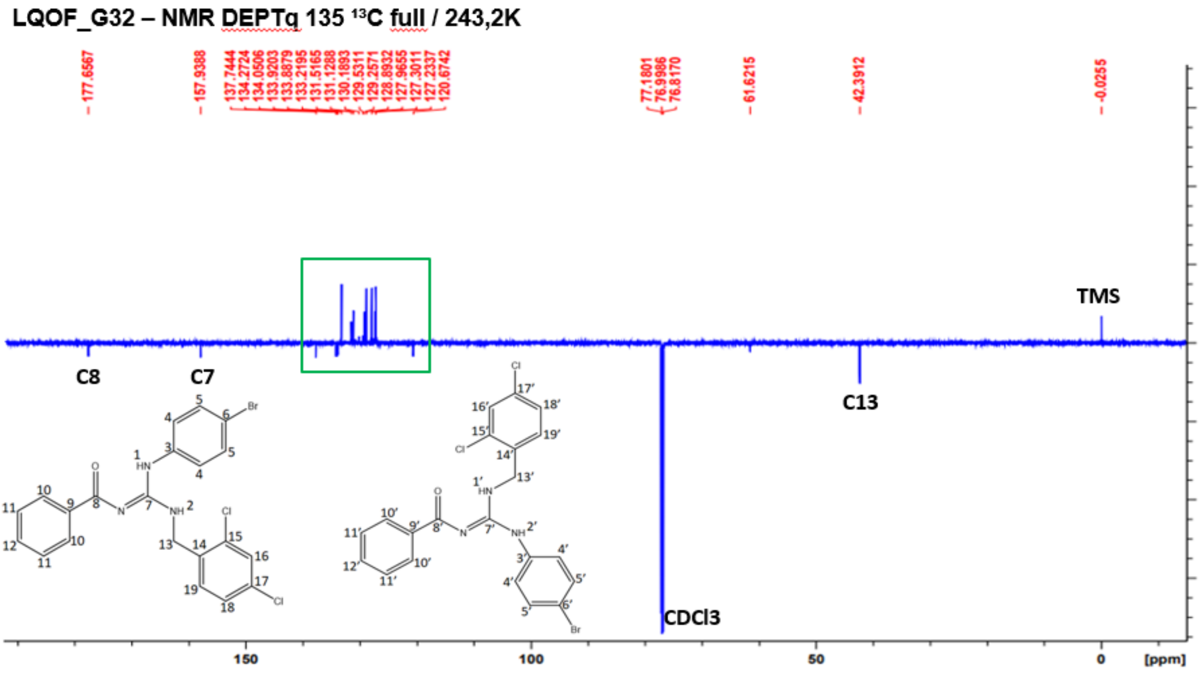

Figure S21. ^{13}C NMR DEPT135q full spectrum of **LQOF-G32** at 243K in $CDCl_3$.

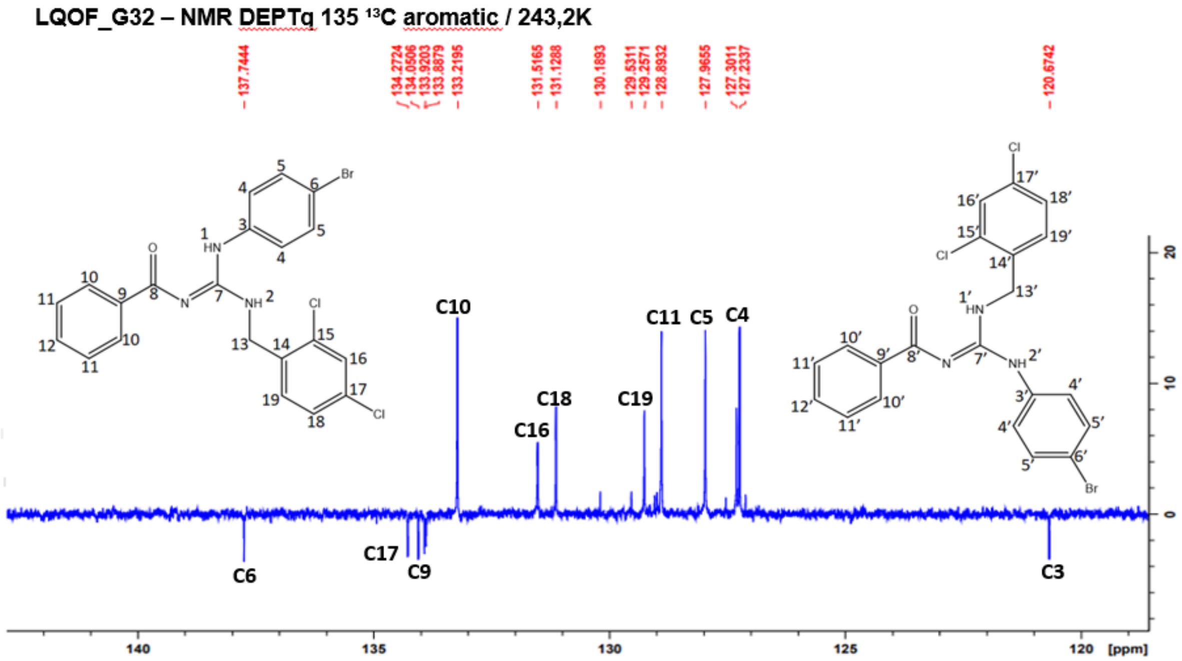

Figure S22. ^{13}C NMR DEPT135q aromatic spectrum of **LQOF-G32** at 243K in $CDCl_3$

LQOF-G32 new batch characterization data: ^{1}H NMR (600.25 MHz, $CDCl_3$) δ = 12.17 (br, NH), 8.26 (d, 2H), 7.58 (d, 2H), 7.54 (t, 1H), 7.48-7.42 (m, 3H), 7.24 (d, 1H), 7.23 (d, 1H), 7.17 (d, 1H), 4.80 (d, 3H), 1.74 (br, NH). ^{13}C NMR (150.95 MHz, $CDCl_3$) δ = 177.65 (C=O), 157.93 (C), 137.74 (C), 134.27 (C), 134.05 (C), 133.92 (C), 133.88 (C), 133.21 (2CH), 131.51 (CH), 131.12 (CH), 129.25 (CH), 128.89 (2CH), 127.96 (2CH), 127.30 (CH), 127.23 (2CH), 120.67 (C), 42.39 (CH_2). HPLC purity 91.45%; mp: 128.1-128.8°C; ESI (+)-MS: *m/z* encontrada 475,9925, *m/z* calculada para [$C_{21}H_{16}BrCl_2N_3O$ + H]$^+$: 475,9927, Fragmento iônico observado em ESI(+)-MS/MS: [M + H – $C_6H_5CONH_2$]$^+$ de *m/z* 356,9373 e [M + H – $C_{13}H_{10}BrCl_2N_2$]$^+$ de *m/z* 122,0601.

DRX

Table S3. **LQOF-G6** Crystal data, data collection parameters, and structure refinement.

Identification code	**LQOF-G6**_Pca21
Empirical formula	$C_{25}H_{27}N_3O$
Formula weight	385.49
Temperature/K	120.0
Crystal system	orthorhombic
Space group	$Pca2_1$
a/Å	12.0522(5)
b/Å	9.4398(3)
c/Å	37.7926(14)
α/°	90
β/°	90
γ/°	90
Volume/Å^3	4299.7(3)
Z	8
ρ_{calc}g/cm^3	1.191
μ/mm^{-1}	0.074
F(000)	1648.0
Crystal size/mm^3	0.2 × 0.06 × 0.02
Radiation	MoKα (λ = 0.71073)
2Θ range for data collection/°	4.314 to 60.128
Index ranges	$-16 \leq h \leq 16$, $-13 \leq k \leq 13$, $-53 \leq l \leq 53$
Reflections collected	140718
Independent reflections	12591 [R_{int} = 0.0427, R_{sigma} = 0.0213]
Data/restraints/parameters	12591/1/529
Goodness-of-fit on F^2	1.023
Final R indexes [I>=2σ (I)]	R_1 = 0.0397, wR_2 = 0.0966
Final R indexes [all data]	R_1 = 0.0476, wR_2 = 0.1015
Largest diff. peak/hole / e Å^{-3}	0.22/-0.19
Flack parameter	-0.1(3)

Datablock: lqof_g6_pca21

Bond precision:	C-C = 0.0032 A		Wavelength=0.71073
Cell:	a=12.0522(5)	b=9.4398(3)	c=37.7926(14)
	alpha=90	beta=90	gamma=90
Temperature: 120 K			

	Calculated	Reported
Volume	4299.7(3)	4299.7(3)
Space group	P c a 21	P c a 21
Hall group	P 2c -2ac	P 2c -2ac
Moiety formula	C25 H27 N3 O	C25 H27 N3 O
Sum formula	C25 H27 N3 O	C25 H27 N3 O
Mr	385.50	385.49
Dx,g cm-3	1.191	1.191
Z	8	8
Mu (mm-1)	0.074	0.074
F000	1648.0	1648.0
F000'	1648.59	
h,k,lmax	16,13,53	16,13,53
Nref	12628[6406]	12591
Tmin,Tmax	0.995,0.999	0.770,0.802
Tmin'	0.985	

Correction method= # Reported T Limits: Tmin=0.770 Tmax=0.802 AbsCorr = MULTI-SCAN

Data completeness= 1.97/1.00 Theta(max)= 30.064

R(reflections)= 0.0397(11132) wR2(reflections)= 0.1015(12591)

S = 1.023 Npar= 529

The following ALERTS were generated. Each ALERT has the format
test-name_ALERT_alert-type_alert-level.
Click on the hyperlinks for more details of the test.

Alert level C

```
PLAT911_ALERT_3_C Missing FCF Refl Between Thmin & STh/L=    0.600            3 Report
```

Alert level G

```
PLAT007_ALERT_5_G Number of Unrefined Donor-H Atoms ..............         4 Report
PLAT032_ALERT_4_G Std. Uncertainty on Flack Parameter Value High .     0.300 Report
PLAT720_ALERT_4_G Number of Unusual/Non-Standard Labels ..........         8 Note
PLAT910_ALERT_3_G Missing # of FCF Reflection(s) Below Theta(Min).         2 Note
PLAT912_ALERT_4_G Missing # of FCF Reflections Above STh/L=  0.600         9 Note
PLAT913_ALERT_3_G Missing # of Very Strong Reflections in FCF ....         1 Note
PLAT955_ALERT_1_G Reported (CIF) and Actual (FCF) Lmax Differ by .         1 Units
PLAT978_ALERT_2_G Number C-C Bonds with Positive Residual Density.        17 Info
```

0 **ALERT level A** = Most likely a serious problem - resolve or explain
0 **ALERT level B** = A potentially serious problem, consider carefully
1 **ALERT level C** = Check. Ensure it is not caused by an omission or oversight
8 **ALERT level G** = General information/check it is not something unexpected

1 ALERT type 1 CIF construction/syntax error, inconsistent or missing data
1 ALERT type 2 Indicator that the structure model may be wrong or deficient
3 ALERT type 3 Indicator that the structure quality may be low
3 ALERT type 4 Improvement, methodology, query or suggestion
1 ALERT type 5 Informative message, check

It is advisable to attempt to resolve as many as possible of the alerts in all categories. Often the minor alerts point to easily fixed oversights, errors and omissions in your CIF or refinement strategy, so attention to these fine details can be worthwhile. In order to resolve some of the more serious problems

it may be necessary to carry out additional measurements or structure refinements. However, the purpose of your study may justify the reported deviations and the more serious of these should normally be commented upon in the discussion or experimental section of a paper or in the "special_details" fields of the CIF. checkCIF was carefully designed to identify outliers and unusual parameters, but every test has its limitations and alerts that are not important in a particular case may appear. Conversely, the absence of alerts does not guarantee there are no aspects of the results needing attention. It is up to the individual to critically assess their own results and, if necessary, seek expert advice.

Publication of your CIF in IUCr journals

A basic structural check has been run on your CIF. These basic checks will be run on all CIFs submitted for publication in IUCr journals (*Acta Crystallographica*, *Journal of Applied Crystallography*, *Journal of Synchrotron Radiation*); however, if you intend to submit to *Acta Crystallographica Section C* or *E* or *IUCrData*, you should make sure that full publication checks are run on the final version of your CIF prior to submission.

Publication of your CIF in other journals

Please refer to the *Notes for Authors* of the relevant journal for any special instructions relating to CIF submission.

PLATON version of 20/01/2022; check.def file version of 19/01/2022

Datablock lqof_g6_pca21 - ellipsoid plot

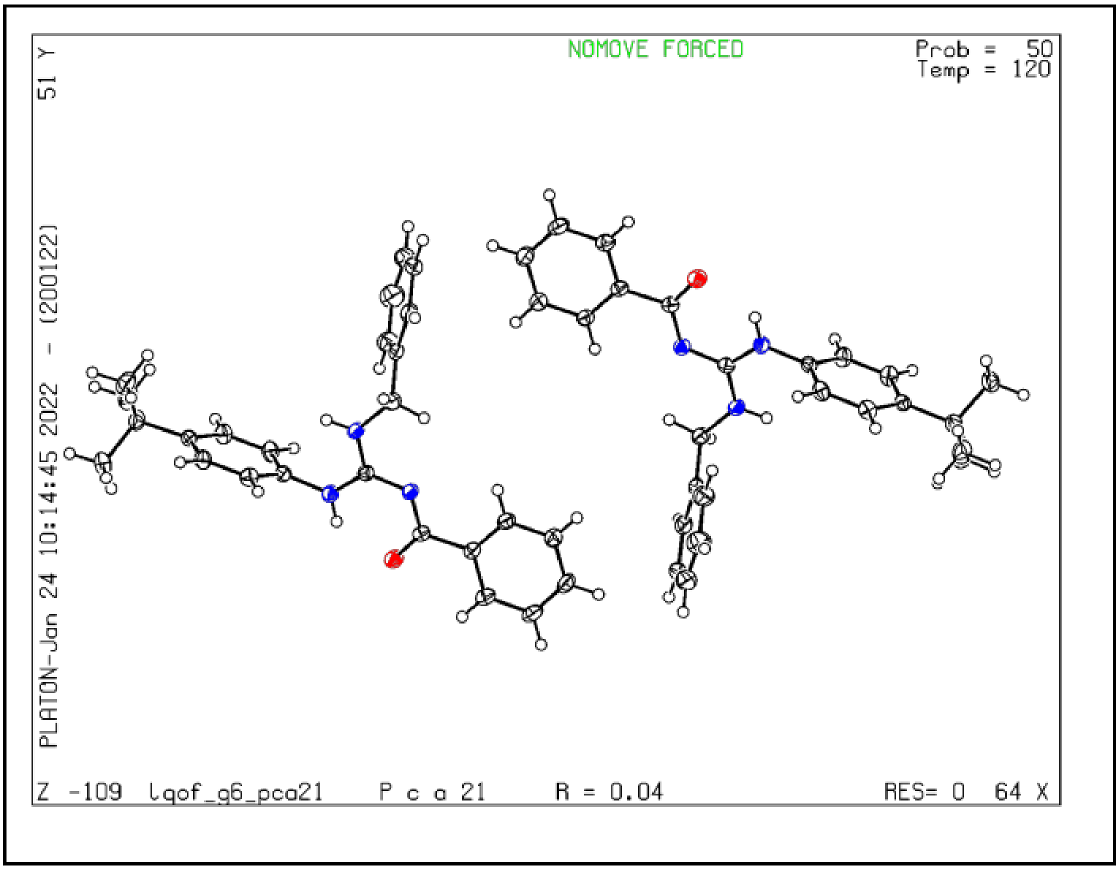

Figure S23. Datablock: lqof_g6_pca21.

Table S4. Sample, crystal data and data collection and structure refinement.

Identification code	**LQOF-G6**_Pca21
Empirical formula	$C_{21}H_{18}N_4O_3$
Formula weight	374.39
Temperature/K	100.0
Crystal system	orthorhombic
Space group	P212121
a/Å	6.9519(6)
b/Å	12.307(2)
c/Å	20.307(2)
α/°	90
β/°	90
γ/°	90
Volume/Å^3	1804.8(3)
Z	4
ρ_{calc}g/cm^3	1.378
μ/mm^{-1}	0.095
F(000)	784.0
Crystal size/mm^3	0.15 × 0.01 × 0.03
Radiation	MoKα (λ = 0.71073)
2Θ range for data collection/°	5.124 to 60.21
Index ranges	-9 ≤ h ≤ 9, -18 ≤ k ≤ 18, -28 ≤ l ≤ 28
Reflections collected	171224
Data/restraints/parameters	5292/0/257
Goodness-of-fit on F^2	1049
Final R indexes [I>=2σ (I)]	R_1 = 0.0337, wR_2 = 0.0853
Final R indexes [all data]	R_1 = 0.0317, wR_2 = 0.0831
Largest diff. peak/hole / e Å^{-3}	0.26/-0.20

No syntax errors found.
Please wait while processing ....

CIF dictionary
Interpreting this report

Datablock: prka_lqof_g1_lt_p212121

Bond precision:	C-C = 0.0021 A		Wavelength=0.71073
Cell:	a=6.9519(6)	b=12.7844(13)	c=20.307(2)
	alpha=90	beta=90	gamma=90
Temperature:	100 K		

	Calculated	Reported
Volume	1804.8(3)	1804.8(3)
Space group	P 21 21 21	P 21 21 21
Hall group	P 2ac 2ab	P 2ac 2ab
Moiety formula	C21 H18 N4 O3	C21 H18 N4 O3
Sum formula	C21 H18 N4 O3	C21 H18 N4 O3
Mr	374.39	374.39
Dx,g cm-3	1.378	1.378
Z	4	4
Mu (mm-1)	0.095	0.095
F000	784.0	784.0
F000'	784.34	
h,k,lmax	9,18,28	9,18,28
Nref	5312[3024]	5292
Tmin,Tmax	0.989,0.997	0.660,0.746
Tmin'	0.986	

Correction method= # Reported T Limits: Tmin=0.660 Tmax=0.746
AbsCorr = MULTI-SCAN

Data completeness= 1.75/1.00   Theta(max)= 30.105

R(reflections)= 0.0317(5085)   wR2(reflections)= 0.0853(5292)

S = 1.049   Npar= 257

The following ALERTS were generated. Each ALERT has the format
test-name_ALERT_alert-type_alert-level.
Click on the hyperlinks for more details of the test.

The following ALERTS were generated. Each ALERT has the format
test-name_ALERT_alert-type_alert-level.
Click on the hyperlinks for more details of the test.

Alert level C

```
STRVA01_ALERT_4_C           Flack parameter is too small
           From the CIF: _refine_ls_abs_structure_Flack    -0.300
           From the CIF: _refine_ls_abs_structure_Flack_su    0.300
PLAT414_ALERT_2_C Short Intra D-H..H-X       H4        ..H7          1.94 Ang.
                                              x,y,z  =       1_555 Check
PLAT480_ALERT_4_C Long H...A H-Bond Reported H15B     ..O2       .       2.61 Ang.
PLAT911_ALERT_3_C Missing FCF Refl Between Thmin & STh/L=    0.600      10 Report
PLAT913_ALERT_3_C Missing # of Very Strong Reflections in FCF ....       4 Note
```

Alert level G

```
PLAT007_ALERT_5_G Number of Unrefined Donor-H Atoms .............        1 Report
PLAT032_ALERT_4_G Std. Uncertainty on Flack Parameter Value High .   0.300 Report
PLAT898_ALERT_4_G Second Reported H-M Symbol in CIF Ignored ......       ! Check
PLAT910_ALERT_3_G Missing # of FCF Reflection(s) Below Theta(Min).       2 Note
PLAT912_ALERT_4_G Missing # of FCF Reflections Above STh/L=  0.600       3 Note
PLAT916_ALERT_2_G Hooft y and Flack x Parameter Values Differ by .    0.12 Check
PLAT933_ALERT_2_G Number of OMIT Records in Embedded .res File ...       2 Note
PLAT978_ALERT_2_G Number C-C Bonds with Positive Residual Density.      16 Info
```

```
 0 ALERT level A = Most likely a serious problem - resolve or explain
 0 ALERT level B = A potentially serious problem, consider carefully
 5 ALERT level C = Check. Ensure it is not caused by an omission or oversight
 8 ALERT level G = General information/check it is not something unexpected

 0 ALERT type 1 CIF construction/syntax error, inconsistent or missing data
 4 ALERT type 2 Indicator that the structure model may be wrong or deficient
 3 ALERT type 3 Indicator that the structure quality may be low
 5 ALERT type 4 Improvement, methodology, query or suggestion
 1 ALERT type 5 Informative message, check
```

It is advisable to attempt to resolve as many as possible of the alerts in all categories. Often the minor alerts point to easily fixed oversights, errors and omissions in your CIF or refinement strategy, so

attention to these fine details can be worthwhile. In order to resolve some of the more serious problems it may be necessary to carry out additional measurements or structure refinements. However, the purpose of your study may justify the reported deviations and the more serious of these should normally be commented upon in the discussion or experimental section of a paper or in the "special_details" fields of the CIF. checkCIF was carefully designed to identify outliers and unusual parameters, but every test has its limitations and alerts that are not important in a particular case may appear. Conversely, the absence of alerts does not guarantee there are no aspects of the results needing attention. It is up to the individual to critically assess their own results and, if necessary, seek expert advice.

Publication of your CIF in IUCr journals

A basic structural check has been run on your CIF. These basic checks will be run on all CIFs submitted for publication in IUCr journals (*Acta Crystallographica*, *Journal of Applied Crystallography*, *Journal of Synchrotron Radiation*); however, if you intend to submit to *Acta Crystallographica Section C* or *E* or *IUCrData*, you should make sure that full publication checks are run on the final version of your CIF prior to submission.

Publication of your CIF in other journals

Please refer to the *Notes for Authors* of the relevant journal for any special instructions relating to CIF submission.

PLATON version of 22/12/2019; check.def file version of 13/12/2019

Datablock prka_lqof_g1_lt_p212121 - ellipsoid plot

Download CIF editor (publCIF) from the IUCr
Download CIF editor (enCIFer) from the CCDC
Test a new CIF entry

Figure S24. Datablock: prka_lqof_g1_It_p212121.

Table S5. Cell viability in cultures growing in the presence of nine different concentrations of **LQOF-G6**.

LQOF-G6 (μM)	HEK-293	KB-3	CACO-2	A-549
1.6	99.7	113.6	105.9	116.3
3.1	101.8	96.9	107.9	108.2
6.2	114.5	103.3	105.5	95.1
12.5	94.05	93.1	95.1	96.2
25	89.9	100.5	102.8	107.6
50	105.4	91.9	104.5	99.2
100	103.8	95.6	103.0	124.3
200	106.6	101.0	110.1	114.8
400	87.2	88.6	83.5	89.6

*Measurement for each concentration was performed in triplicate and the average value is reported.

Table S6. Effect of **LQOF-G6** on the rate of activity of the right atrium.

LQOF-G6 (μM)	f ± SEM* (beats/min)	Δf ± SEM* (%)	Number of Experiments (n)	Probability (*P*)
Control	195.00 ± 21.21	0.00 ± 0.00	4	--
3	188.75 ± 21.22	-3.52 ± 1.58	4	n.s.
10	182.50 ± 23.24	-7.12 ± 2.70	4	n.s.
30	178.75 ± 21.93	-8.70 ± 3.85	4	n.s.
100	172.50 ± 22.78	-12.31 ± 4.24	4	<0.05

Table S7. Effect of **LQOF-G6** on the contraction force of the papillary muscle.

LQOF-G6 (μM)	f_c ± SEM* (mN)	Δf_c ± SEM* (%)	Number of Experiments (n)	Probability (*P*)
Control	1.32 ± 0.21	0.00 ± 0.00	4	--
3	1.20 ± 0.21	-9.27 ± 5.91	4	n.s.
10	1.21 ± 0.14	-5.41 ± 6.95	4	n.s.
30	1.20 ± 0.14	2.18 ± 6.64	4	n.s.
100	1.35 ± 0.14	5.43 ± 6.54	4	n.s.

Table S8. Effect of **LQOF-G6** on the contraction force of the aorta.

LQOF-G6 (μM)	f_c ± SEM* (mN)	Δf_c ± SEM* (%)	Number of Experiments (n)	Probability (P)
Control	10.34 ± 0.91	0.00 ± 0.00	5	--
3	10.23 ± 0.91	-1.11 ± 2.27	5	n.s.
10	10.09 ± 0.93	-2.39 ± 3.37	5	n.s.
30	9.79 ± 0.86	-5.17 ± 3.91	5	n.s.
100	9.37 ± 0.84	-9.06 ± 4.90	5	n.s.

Table S9. Effect of **LQOF-G6** on the contraction force of the arteria pulmonalis.

LQOF-G6 (μM)	fc ± SEM* (mN)	Δfc ± SEM* (%)	Number of Experiments (n)	Probability (P)
Control	9.45 ± 0.77	0.00 ± 0.00	5	--
3	8.42 ± 0.83	-11.13 ± 3.42	5	<0.05
10	7.91 ± 0.91	-16.90 ± 4.28	5	<0.05
30	7.04 ± 0.96	-26.45 ± 5.30	5	<0.05
100	5.61 ± 1.08	-41.75± 7.93	5	<0.01

Table S10. Effect of **LQOF-G6** on the contraction force of the terminal ileum.

LQOF-G6 (μM)	f_c ± SEM* (mN)	Δf_c ± SEM* (%)	Number of Experiments (n)	Probability (P)
Control	8.71 ± 1.35	0.00 ± 0.00	5	--
3	7.12 ± 1.41	-20.02 ± 4.03	5	< 0.05
10	4.92 ± 1.42	-45.57 ± 8.84	5	<0.01
30	3.54 ± 1.19	-60.07 ± 9.04	5	<0.01
100	2.93 ± 1.00	-67.99 ± 6.67	5	<0.01

Docking Investigation

LQOF-G1 Molecule

The simulation performed upon the subunit A yielded the ligand-protein interactions described in Table S10, where the lower energy conformation is showed in the Figure S14(a) 3D representation and Figure S14(b) 2D representation.

Table S11. Conformations resulted from the docking procedure, ligand-target affinity with its deviation. RMSD = Root mean square deviation; l.b = lower bound; u.b = upper bound.

		Distance from the "best" mode	
**Mode**	**Affinity (kcal/mol)**	**RMSD l.b.**	**RMSD u.b.**
1	-7.9	0.000	0.000
2	-7.7	1.536	2.162
3	-7.6	5.907	11.58
4	-7.5	6.007	11.54
5	-7.2	4.897	10.50
6	-7.2	7.397	10.43
7	-7.1	6.065	9.727
8	-7.1	6.666	12.12
9	-7.0	5.528	10.71

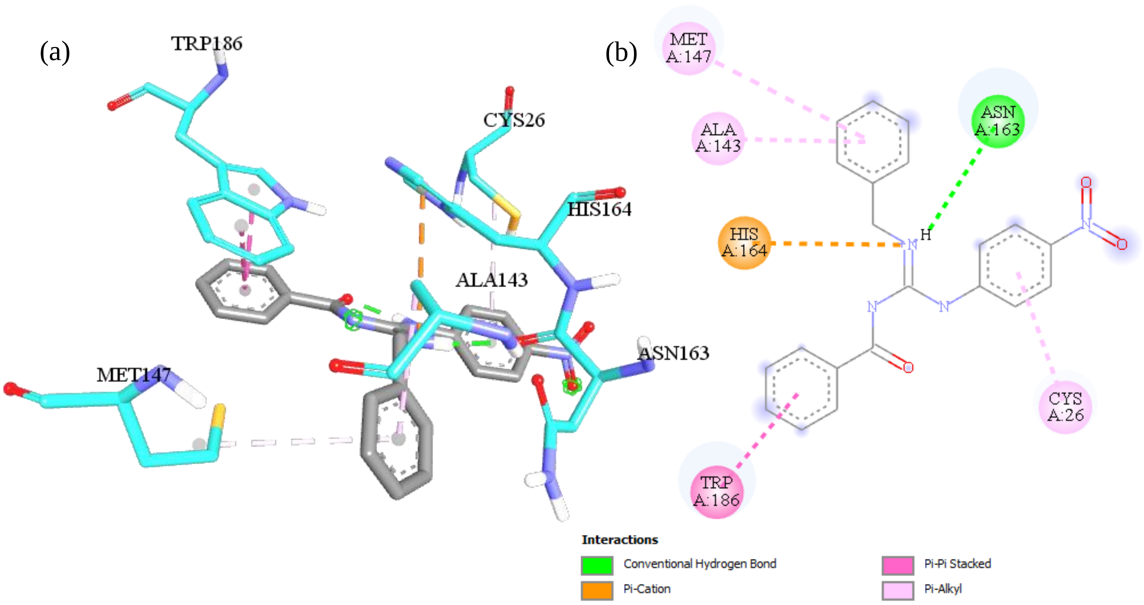

Figure S25. Interactions between the aminoacid residues with the conformation of higher affinity in (a) 3D representation; (b) 2D representation. Cyan = carbon, red = oxygen, purple = nitrogen, yellow = sulfur, white = hydrogen. For the LQOF-G1 molecule.

The residues MET147, ALA143, and CYS26 possess the same type of interaction (π-alkyl) starting from π-orbitals with distances of 5.1593 Å, 4.6389 Å, and 4.8929 Å, respectively. The presence of the nitro electron-withdrawing group directly affects the interaction with the π-orbitals due to the weakening of ring and ligand synergy. This effect was elucidated from the quantum chemical DFT calculations arising out of the isosurface of the active orbitals. Figure S15 shows the representations of the molecular orbitals, where we can observe that the localization of the electron density is upon the nitro group and ring in the LUMO and LUMO+1 orbital. This translates into higher energy of the interaction with CYS26 residue, decreasing the affinity.

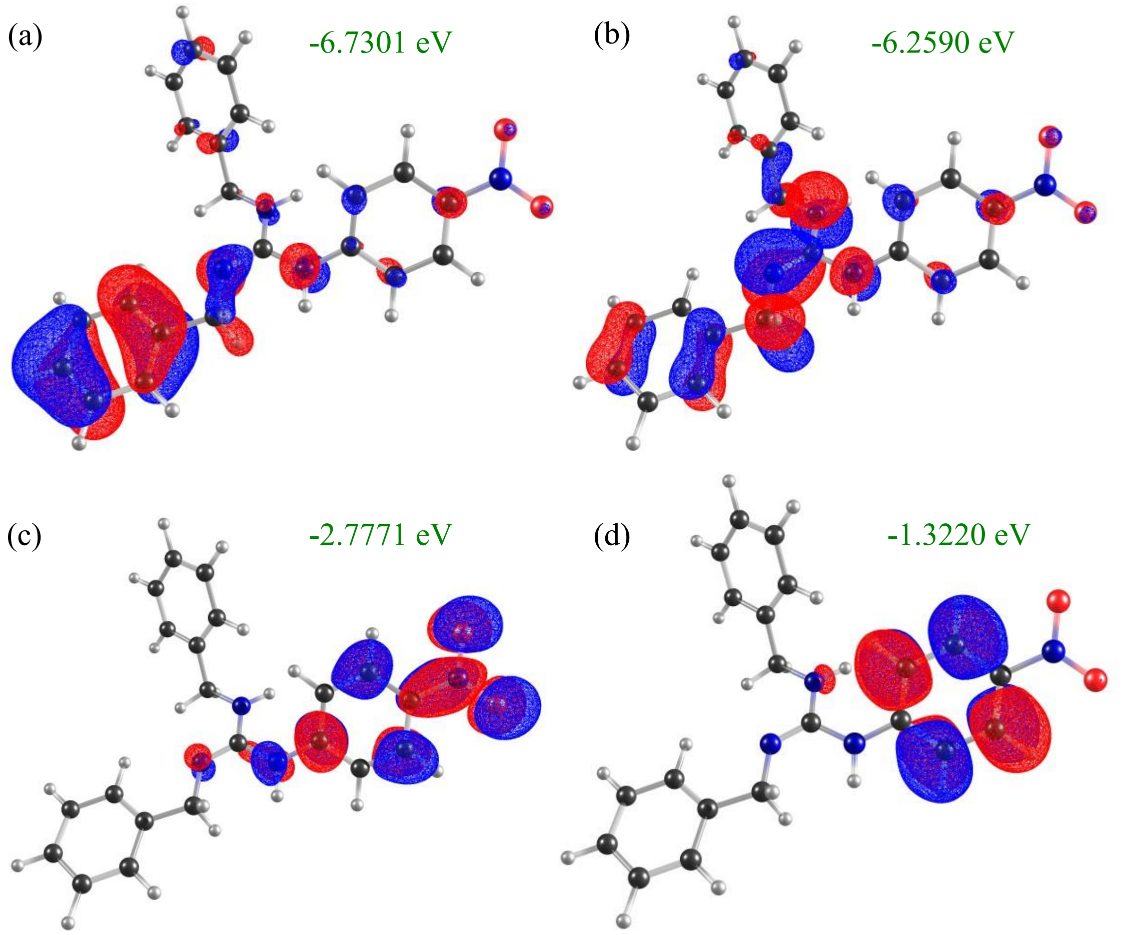

Figure S26. Representation of the molecular orbitals (a) HOMO-1, (b) HOMO, (c) LUMO, (d) LUMO+1 for the LQOF-G1.

LQOF-G2 Molecule

From the docking simulation performed with the same procedure of the LQOF-G1 molecule, nine conformations were yielded with distinct interaction affinity, described in Table S11, where the lower energy conformation is showed in the Figure S16(a) 3D representation and Figure S16(b) 2D representation.

Table S12. Conformations resulted from the docking procedure, ligand-target affinity with its deviation. RMSD = Root mean square deviation; l.b = lower bound; u.b = upper bound.

		Distance from the "best" mod	
**Mode**	**Affinity (Kcal/mol)**	**RMSD l.b.**	**RMSD u.b.**
1	-8.3	0.000	0.000
2	-8.1	1.405	1.884
3	-7.6	7.588	11.43
4	-7.5	6.386	11.23

5	-7.2	6.780	11.44
6	-6.9	6.380	11.15
7	-6.7	7.585	11.35
8	-6.7	10.75	14.19
9	-6.6	3.098	5.756

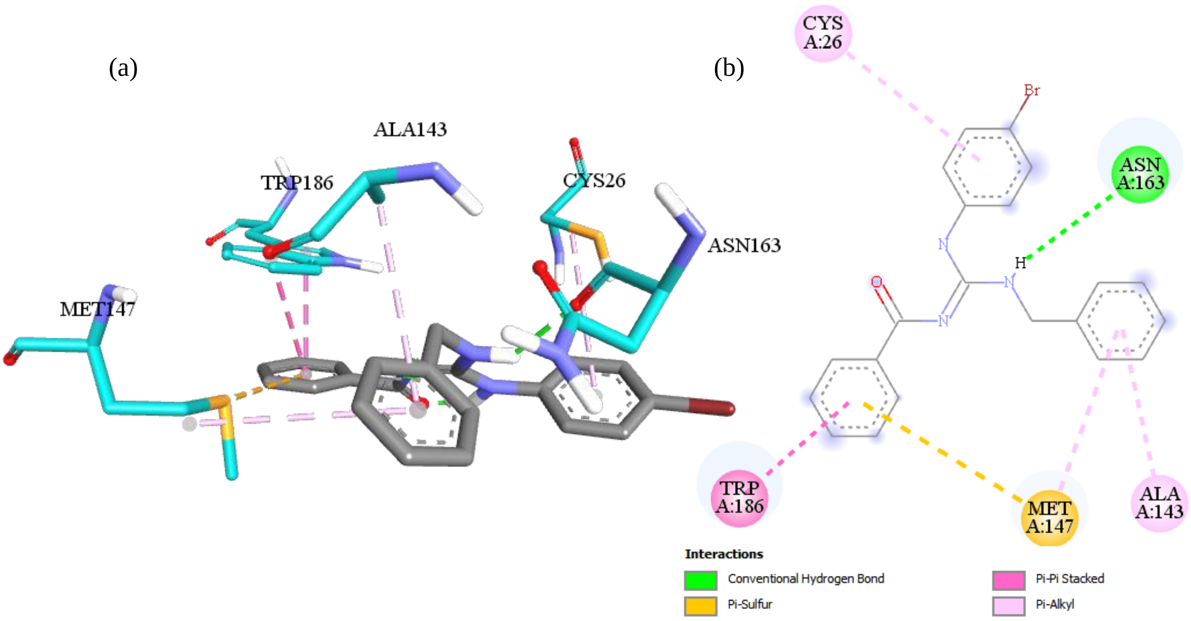

Figure S27. Interactions between the aminoacid residues with the conformation of higher affinity in (a) 3D representation; (b) 2D representation. Cyan = carbon, red = oxygen, purple = nitrogen, yellow = sulfur, white = hydrogen, wine = bromine. For the LQOF-G2 molecule.

From the docking results, the ALA143 and CYS26 possess the same type of interaction with the ring, i.e, π-alkyl starting from the π-orbitals with distances of 4.6406 Å and 5.1131 Å, for each residue, respectively. In comparison with the LQOF-G1 molecule, there was an increase in the distance with CYS26 residue, assigned to the presence of Br atom for being an electron-withdrawing ligand from the inductive effect and electon-donating from resonant effect. Additionally, we can notice the competing a π-sulfur interaction between the ME147 and the amidic ring with the π-π stacked interaction with the benzylamine. The isosurfaces of the active molecular orbitals for this molecule were also elucidated from the quantum mechanical calculations on the DFT level. From Figure S17, in contrast to the LQOF-G1, the HOMO possesses the electron

density upon the p-bromine ring alongside LUMO and LUMO+1. This observation highlights the electron-donating character of bromine atom, decreasing the energy of the π-orbitals that will interact with CYS26 residue, increasing the affinity.

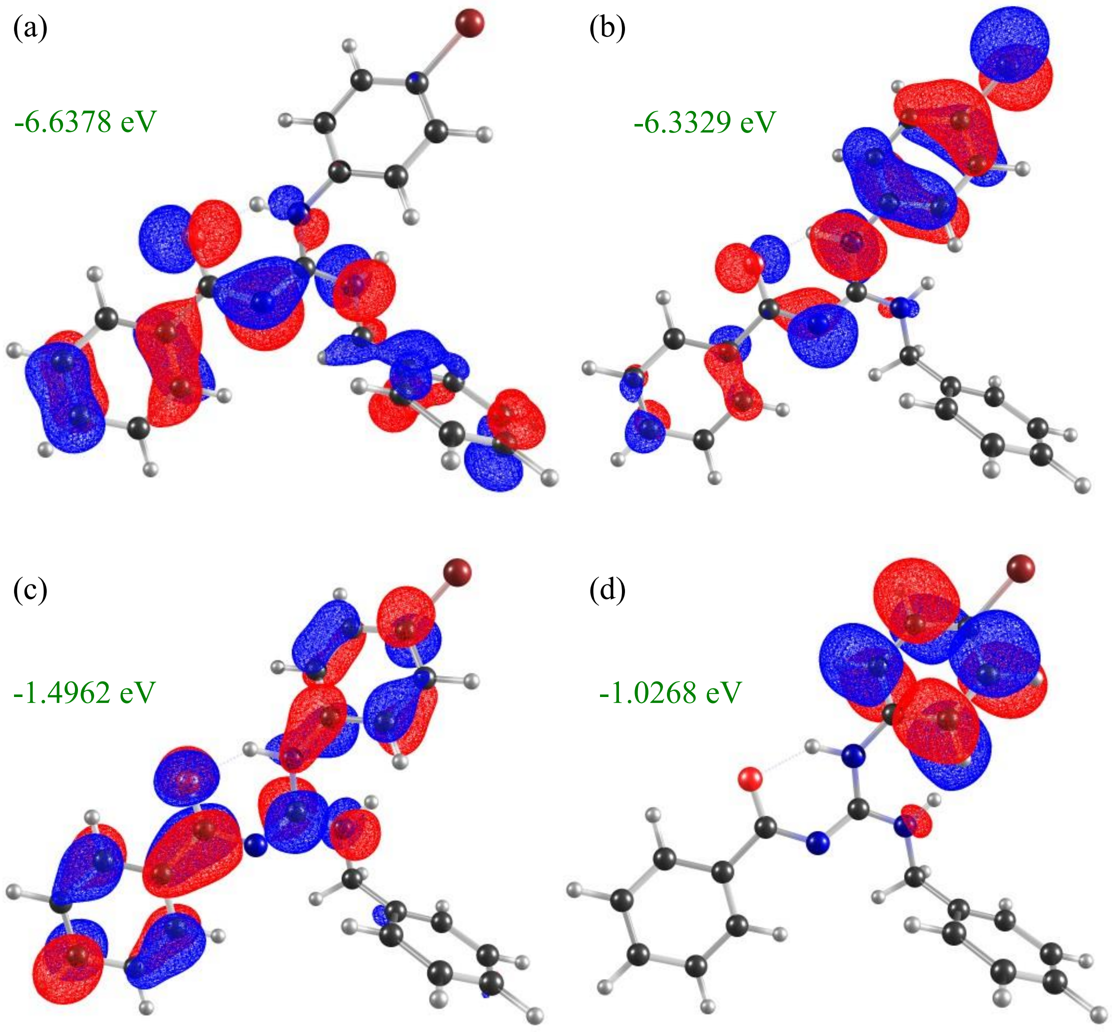

Figure S28. Representation of the molecular orbitals (a) HOMO-1, (b) HOMO, (c) LUMO, (d) LUMO+1. For the LQOF-G2 molecule.

LQOF-G6

From the molecular docking performed with the same procedure of the previous molecules, nine conformations with different ligand-target affinity were yielded, which are described in the Table S12, where the lower energy conformation is showed in Figure S18(a) 3D representation and Figure S18(b) 2D representation.

Table S13. Conformations resulted from the docking procedure, ligand-target affinity with its deviation. RMSD = Root mean square deviation; l.b = lower bound; u.b = upper bound.

		Distance from the "best" mode	
**Mode**	**Affinity (Kcal/mol)**	**RMSD l.b.**	**RMSD u.b.**
1	-8.8	0.000	0.000
2	-8.6	0.929	2.045
3	-8.5	1.357	7.789
4	-7.9	1.758	4.748
5	-7.6	1.579	4.678
6	-7.2	3.101	6.831
7	-7.1	1.728	4.239
8	-7.0	1.496	7.725
9	-6.8	2.562	3.453

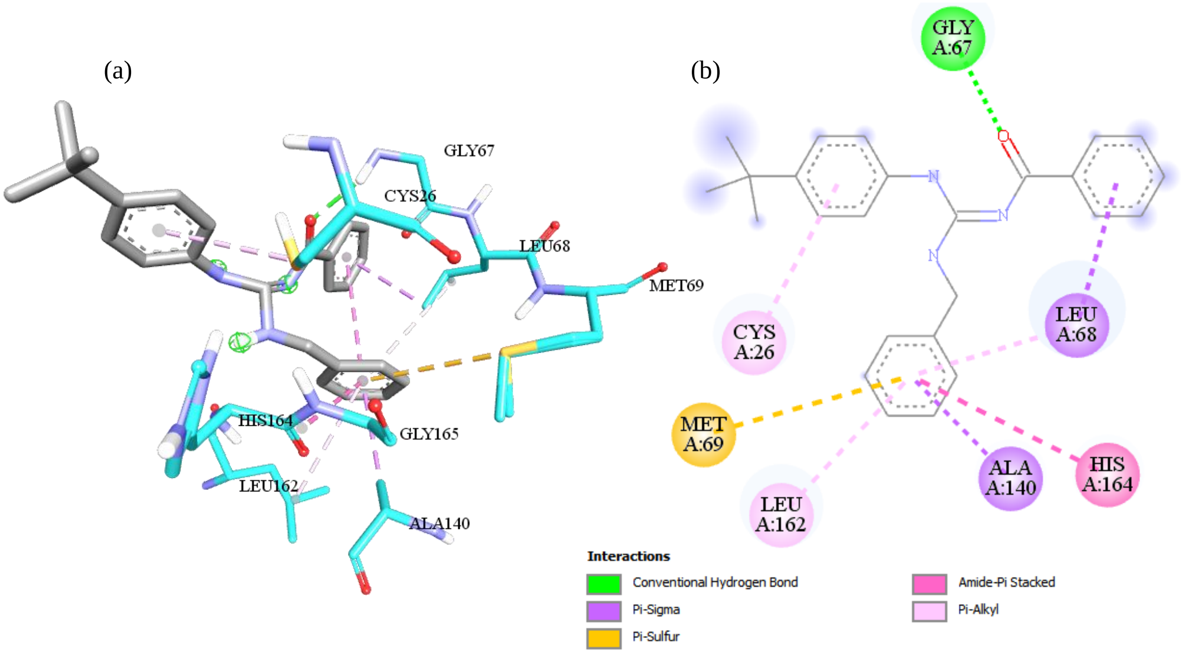

Figure S29. Interactions between the aminoacid residues with the conformation of higher affinity in (a) 3D representation; (b) 2D representation. Cyan = carbon, red = oxygen, purple = nitrogen, yellow = sulfur, white = hydrogen. For the LQOF-G6 molecule

In the LQOF-G6 molecule, we can notice a novel interaction between the upper-right side aromatic ring with LEU68 from a π-sigma mechanism and LEU162 with the lower-centered aromatic ring through a π-alkyl process. Additionally, the CYS26, LEU68, and LEU162 residues exhibit interactions of the same type, starting from the π-

orbitals with distances of 5.3806 Å, 5.3776 Å, and 5.0867 Å, respectively. The increase in the distance between CYS26 residue and the ring can be assigned to the electron-donating character of the tert-butyl group, which is accordance with the observations for the LQOF-G1 and LQOF-G2 molecules. The positions of the electron densities were obtained from a quantum mechanical calculation on the DFT level (Figure S19). The results indicate that although the tert-butyl is not directly contributing to the formation of the active molecular orbitals, its presence induces a modification of the electron density. Here, the isosurfaces are localized upon HOMO-1 and HOMO molecular orbitals instead of LUMO-type orbitals. Due to the lower energy of the HOMO orbitals, there is an increase in the affinity of the π-alkyl interactions.

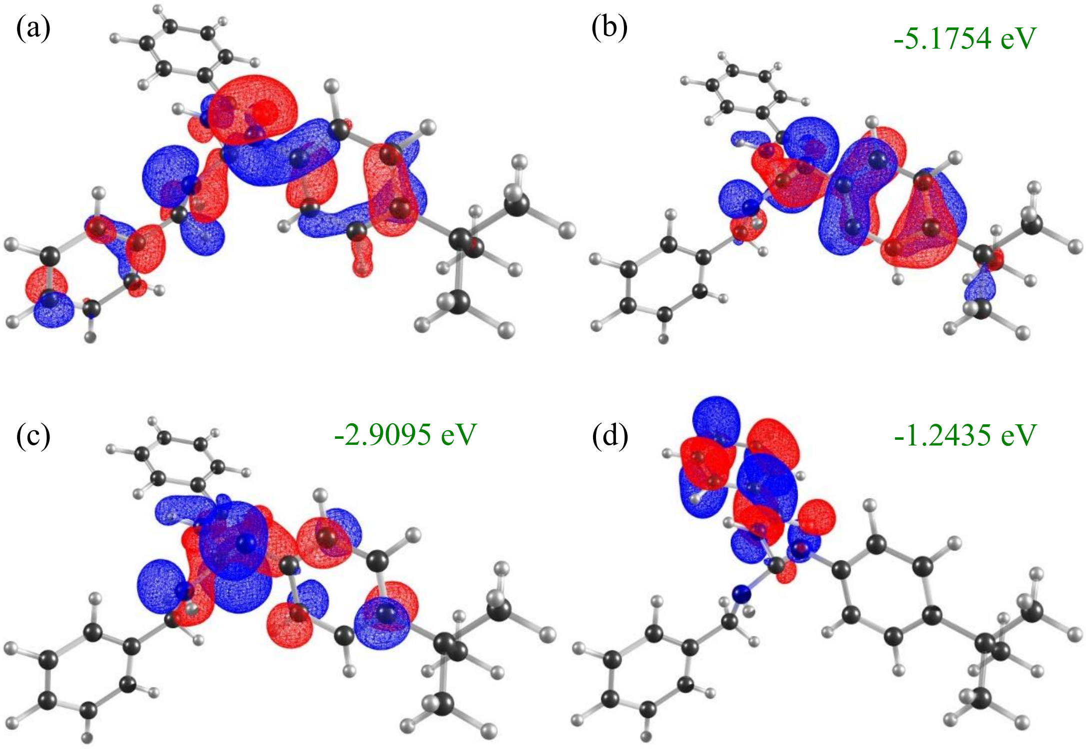

Figure S30. Representation of the molecular orbitals (a) HOMO-1, (b) HOMO, (c) LUMO, (d) LUMO+1 for the LQOF-G6 molecule.

LQOF-G32

The simulation performed upon the subunit A yielded the ligand-protein interactions described in Table S13, where the lower energy conformation is showed in the Figure S20(a) 3D representation and Figure S20(b) 2D representation.

Table S14. Conformations resulted from the docking procedure, ligand-target affinity with its deviation. RMSD = Root mean square deviation; l.b = lower bound; u.b = upper bound.

		Distance from the "best" mode	
**Mode**	**Affinity (Kcal/mol)**	**RMSD l.b.**	**RMSD u.b.**
1	-8.2	0.000	0.000
2	-8.1	10.40	12.92
3	-8.0	7.658	10.55
4	-7.8	3.558	7.407
5	-7.6	3.776	7.294
6	-7.6	7.280	9.509
7	-7.5	9.573	12.04
8	-7.2	6.580	9.366
9	-6.9	4.116	7.005

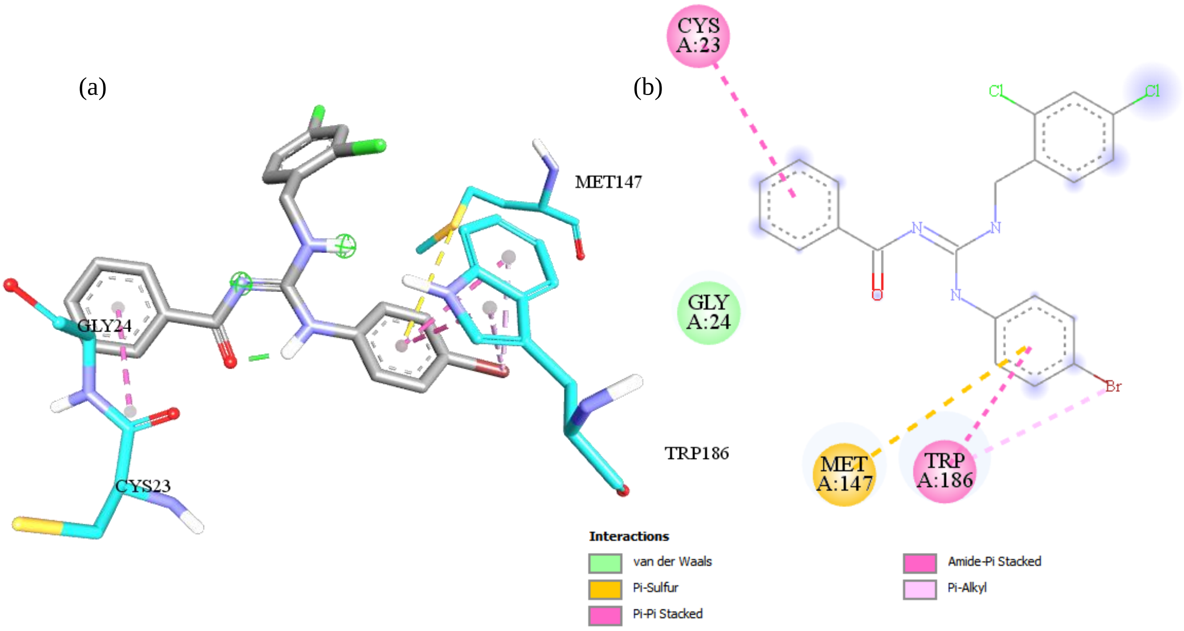

Figure S31. Interactions between the aminoacid residues with the conformation of higher affinity in (a) 3D representation; (b) 2D representation. Cyan = carbon, red = oxygen, purple = nitrogen, yellow = sulfur, white = hydrogen, wine = bromine, green = chlorine.

For the LQOF-G32 molecule.

From Figure S20(a) and (b), we notice the interactions of TRP186 and MET147 residues with the bromobenzyl ring through a π-π stacked and π-sulfur mechanism, respectively. Also, there is competition from TRP186 residue with the bromine atom from a π-alkyl process. The distances for both residues are 3.9224 Å and 5.2821 Å respectively, while CYS23 aminoacid interacts with the amidic ring via an amide-π stacked mechanism with 4.5842 Å. The difference between this distance from the previous for a CYS residue arises from a distinct interaction mechanism. The influence of the chlorine and bromine atoms on the molecular orbitals were also elucidated from quantum mechanical calculation on the DFT level (Figure S21). We notice that Cl atoms directly affects the formation of the LUMO and LUMO+1, due to their contribution to the formation of these MOs. Additionally, there is a redistribution of electron density occurring from the HOMO to LUMO orbitals.

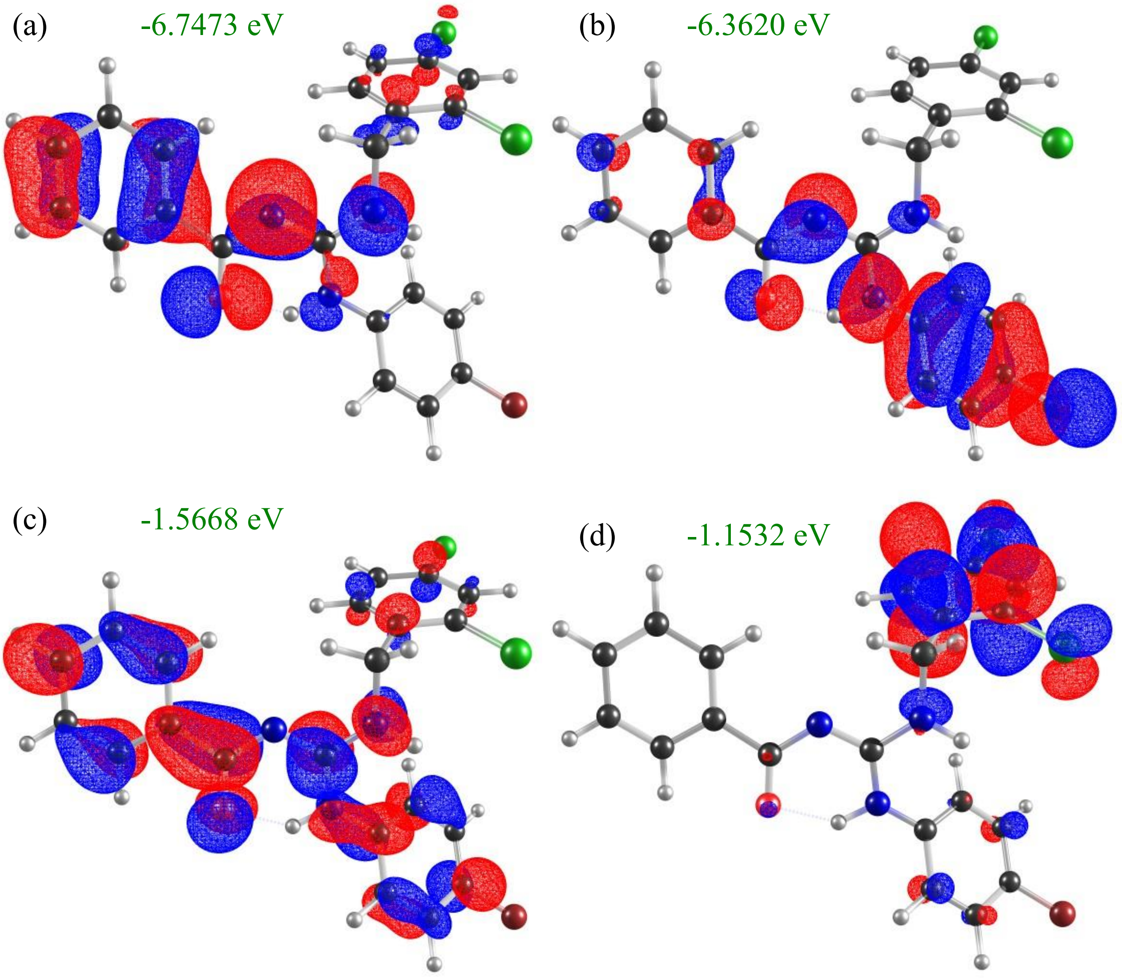

Figure S32. Representation of the molecular orbitals (a) HOMO-1, (b) HOMO, (c) LUMO, (d) LUMO+1 for the LQOF-G32 molecule.

1. STUDY REFERENCES

**Study title**	***In Vitro* Pharmacology: Proteases Assays**<br>**Study of LQOF-G6**	
**Study number**	**100062592**	**FINAL REPORT**<br>**May 10, 2022**
**Study ID**	**FR095-0032854**	
**Experimental period**	May 3, 2022 - May 10, 2022	

2. PERSONS INVOLVED IN THE STUDY

**Technical contact**	**Eurofins Cerep**<br>Le Bois l'Evêque<br>B.P. 30001<br>86 600 Celle l'Evescault<br>France<br>Tel: +33 (0)5 49 89 30 00<br>Fax: +33 (0)5 49 43 21 70	**Carsten BROCK,Ph.D.**<br>Study Director<br>CarstenBrock@eurofins.com
**Study sponsor**	**University of Vienna**<br>Dept of Pharm Chemistry<br>1090 Vienna<br>AUSTRIA	**Dr. Predrag KALABA**

3. APPROVAL

**Head of laboratory statement**	This study was conducted according to the procedures described in this report.<br><br>**Eurofins Cerep**<br>Le Bois l'Evêque<br>B.P. 30001<br>86 600 Celle l'Evescault<br>France	 <br>**Sophie SEIGNEURIN,Mrs**<br>Operations Director<br>SophieSeigneurin@eurofins.com<br><br>Signature
**Quality assurance statement**	This study was inspected by Eurofins Cerep Quality Control Unit, the results and methods presented in this report accurately reflect the methods used and the data collected for this study.<br><br>**Eurofins Cerep**<br>Le Bois l'Evêque<br>B.P. 30001<br>86 600 Celle l'Evescault<br>France	 <br><br><br>**Eric BOUCHET**<br>Quality Site Group Leader<br>EricBouchet@eurofins.com<br><br>Signature

4. TABLE OF CONTENTS

1. STUDY REFERENCES 2
2. PERSONS INVOLVED IN THE STUDY 2
3. APPROVAL 3
4. TABLE OF CONTENTS 4
5. SUMMARY 5
5.1. Study Design 5
5.2. Measurements 5
5.3. Results 5
6. COMPOUNDS 6
6.1. Test Compounds 6
6.2. Reference Compounds 6
7. RESULTS 7
7.1. *In Vitro* Pharmacology: Enzyme and Uptake Assays 7
7.1.1. Test Compound Results 7
7.1.2. Reference Compound Results 7
8. RESULTS INTERPRETATION GUIDE 8
9. MATERIALS AND METHODS 9
9.1. Experimental Conditions 9
9.1.1. *In Vitro* Pharmacology: Enzyme and Uptake Assays 9
9.2. Analysis and expression of results 10
9.2.1. *In Vitro* Pharmacology: Enzyme and Uptake Assays 10
10. BIBLIOGRAPHY 11

5. SUMMARY

The purpose of this study was to test LQOF-G6 in enzyme and uptake assays.

5.1. Study Design

LQOF-G6 was tested at 1.0E-06 M and 1.0E-05 M.

5.2. Measurements

Compound enzyme inhibition effect was calculated as a % inhibition of control enzyme activity.

5.3. Results

Results showing an inhibition or stimulation higher than 50% are considered to represent significant effects of the test compounds.

Such effects were not observed at any of the receptors studied.

6. COMPOUNDS

6.1. Test Compounds

Client Compound ID	Compound ID	Reference Number	Batch Number	FW	MW	Purity	Received Form	Stock solution	Flag
LQOF-G6	100062592-2	-	-	385.22	-	100.0	Liquid	1.E-02 M DMSO	-

FW: Formula Weight - MW: Molecular Weight

6.2. Reference Compounds

In each experiment and if applicable, the respective reference compound was tested concurrently with LQOF-G6, and the data were compared with historical values determined at Eurofins. The experiment was accepted in accordance with Eurofins validation Standard Operating Procedure.

7. RESULTS

7.1. *In Vitro* Pharmacology: Enzyme and Uptake Assays

7.1.1. Test Compound Results

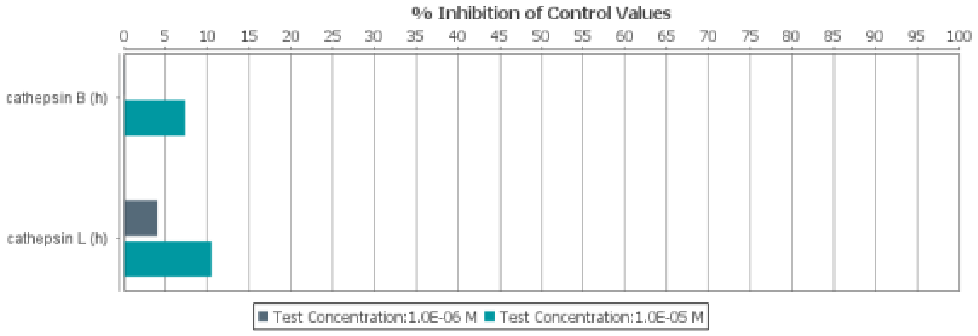

Figure 1. Histogram for LQOF-G6

Compound I.D.	Client Compound I.D.	Test Concentration	% Inhibition of Control Values 1st	2nd	Mean
**cathepsin B *(h)***					
100062592-2	LQOF-G6	1.0E-06 M	0.3	-0.2	0.1
100062592-2	LQOF-G6	1.0E-05 M	8.0	6.5	7.3
**cathepsin L *(h)***					
100062592-2	LQOF-G6	1.0E-06 M	-0.8	8.7	4.0
100062592-2	LQOF-G6	1.0E-05 M	12.3	8.7	10.5

7.1.2. Reference Compound Results

Compound I.D.	IC_{50}(M)	nH
**cathepsin B *(h)***		
leupeptin	1.7E-08 M	0.9
**cathepsin L *(h)***		
leupeptin	2.1E-09 M	1.1

8. RESULTS INTERPRETATION GUIDE

In Vitro Pharmacology

Results showing an inhibition (or stimulation for assays run in basal conditions) higher than 50% are considered to represent significant effects of the test compounds. 50% is the most common cut-off value for further investigation (determination of IC_{50} or EC_{50} values from concentration-response curves) that we would recommend.
Results showing an inhibition (or stimulation) between 25% and 50% are indicative of weak to moderate effects (in most assays, they should be confirmed by further testing as they are within a range where more inter-experimental variability can occur).
Results showing an inhibition (or stimulation) lower than 25% are not considered significant and mostly attributable to variability of the signal around the control level.
Low to moderate negative values have no real meaning and are attributable to variability of the signal around the control level. High negative values (≥ 50%) that are sometimes obtained with high concentrations of test compounds are generally attributable to non-specific effects of the test compounds in the assays. On rare occasion they could suggest an allosteric effect of the test compound.

9. MATERIALS AND METHODS

9.1. Experimental Conditions

Minor variations to the experimental protocol described below may have occurred during the testing, they have no impact on the quality of the results obtained.

9.1.1. *In Vitro* Pharmacology: Enzyme and Uptake Assays

Assay	Source	Substrate/ Stimulus/Tracer	Incubation	Measured Component	Detection Method	Bibl.
**Other enzymes**						
**cathepsin B *(h)***	human placenta	Z-Arg-Arg-pNa (0.3 mM)	180 min 37 °C	pNa	Photometry	10
**cathepsin L *(h)***	human liver	Z-Phe-Arg-AMC (15 µM)	30 min RT	AMC	Fluorimetry	10

9.2. Analysis and expression of results

9.2.1. *In Vitro* Pharmacology: Enzyme and Uptake Assays

The results are expressed as a percent of control specific activity

$$\frac{\text{measured specific activity}}{\text{control specific activity}} * 100$$

and as a percent inhibition of control specific activity

$$100 - \left(\frac{\text{measured specific activity}}{\text{control specific activity}} * 100\right)$$

obtained in the presence of LQOF-G6.

The IC_{50} values (concentration causing a half-maximal inhibition of control specific activity), EC_{50} values (concentration producing a half-maximal increase in control basal activity), and Hill coefficients (nH) were determined by non-linear regression analysis of the inhibition/concentration-response curves generated with mean replicate values using Hill equation curve fitting

$$Y = D + \left[\frac{A - D}{1 + (C/C_{50})^{nH}}\right]$$

where Y = specific activity, A = left asymptote of the curve, D = right asymptote of the curve, C = compound concentration, C_{50} = IC_{50} or EC_{50} , and nH = slope factor.
This analysis was performed using software developed at Cerep (Hill software) and validated by comparison with data generated by the commercial software SigmaPlot® 4.0 for Windows® (© 1997 by SPSS Inc.).

10. BIBLIOGRAPHY

10. Barrett, A.J. and Kirschke, H. (1981), *Meth. Enzymol.*, 80: 535-561.

Figure S33. In Vitro Pharmacology: Proteases Assays Report.
